# Supplementary material for: Uncoupling key determinants of hematopoietic stem cell engraftment through cell-specific and temporally controlled recipient conditioning
Source: Stem Cell Reports. 2021 Jun 24;16(7):1705–17. doi: 10.1016/j.stemcr.2021.05.019 (PMC8282468; doi:10.1016/j.stemcr.2021.05.019)
Supplement: Document S1. Figures S1–S4 [file mmc1.pdf]

Stem Cell Reports, Volume *16*

Supplemental Information

Uncoupling key determinants of hematopoietic stem cell engraftment through cell-specific and temporally controlled recipient conditioning

Natsumi Miharada, Anna Rydström, Justyna Rak, and Jonas Larsson

Supplemental Information

Uncoupling key determinants of hematopoietic stem cell engraftment through cell specific and temporally controlled recipient conditioning

Natsumi Miharada *et al.*

Figure S1, related to Figure 1. Conditional *Gata2* deletion depletes phenotypic and functional HSCs

(A) Genomic PCR results with unfractionated BM from ER-Cre^{+} *Gata2*$^{fl/fl}$ and control (ER-Cre^{-} *Gata2*$^{fl/fl}$) mice administrated with tamoxifen and harvested at 1 week after the last injection. (B) Representative FACS plots showing LSK compartment at 1 day after the last tamoxifen injection. (C) Frequency (left) and cell number (right) of LSK from ER-Cre^{+} *Gata2*$^{fl/fl}$ and control mice (ER-Cre^{-} *Gata2*$^{fl/fl}$) at 1-7 day after the last tamoxifen injection. Data are pooled from three independent experiments (n = 10). (D and E) BM (Ly5.2) from ER-Cre^{+} *Gata2*$^{fl/fl}$ mice that received 5 injections of tamoxifen or vehicle were transplanted together with WT BM (Ly5.1) cells into irradiated recipients in a competitive manner (n = 6-7). Frequency of Ly5.2 cells in PB (D) and BM (E) after the transplantation. Representative FACS plots showing the contribution from each donor at 20 weeks after transplantation (n = 6-7). n represents independent biological replicates, except in D and E where n represents technical replicates (recipient mice) from 3 independent biological repeats (donor mice).

Figure S2, related to Figure 2. *Gata2* deletion enables efficient engraftment of transplanted HSCs

(A and B) Frequency of donor derived cells within each lineages in PB after the transplantation, corresponding to Figure 2B and 2E respectively. (C) 70,000 LSK cells were transplanted into ER-Cre^{+} *Gata2*$^{fl/fl}$ or control (ER-Cre^{-} *Gata2*$^{fl/fl}$) mice. 4 weeks after the transplantation, recipients were treated with tamoxifen for 5 injections. (D) Donor contribution in PB was monitored for up to 22 weeks after the last tamoxifen injection (n = 3-4). (E) Frequency of donor in BM LSK at 22 weeks after the last tamoxifen injection (n = 3-4). n represents independent biological replicates.

Figure S3, related to Figure 2. Detection of donor engraftment in non-conditioned recipients

(A) 320,000 LSK cells from WT (Ly5.2) were transplanted into WT recipients (Ly5.1/5.2 F1) without irradiation. After 4 weeks, total BM cells of primary recipients were harvested, and 5 million cells were further transplanted into lethally irradiated secondary WT recipients (Ly5.1/5.2 F1). (B) Frequency of donor derived cells in the BM HSC at 4 weeks after the primary transplantation (n = 3). FACS plots showing the gating strategy for the non-transplanted control mouse (above) and primary recipient (below). (C) Frequency of initial donor derived cells in the peripheral blood of secondary recipients (BM from 3 primary recipients was transplanted to 2 secondary recipients each). n represents independent biological replicates.

Figure S4, related to Figure 4. Advantage in homing is associated with higher engraftment potential in non-irradiated recipients

(A) Relative mRNA expression of indicated genes normalized to *Hprt* (LSK n = 9, HSC n = 5). (B) As a reference for the initial ratio of Ly5.1 (labelled with CFSE) and Ly5.2 (labelled with SNARF-1) cells before the transplantation, some residues of the transplanted cells were kept on ice and analyzed at the same time with the homed cells to the BM in the recipients. (C) Gating strategy to record statistically relevant numbers of CFSE and SNARF-1 positive cells homed to the bone marrow. After doublets and dead cell exclusion, unstained and autofluorescent cells were excluded as an inverted gate. n represents independent biological replicates.

Figure S1.

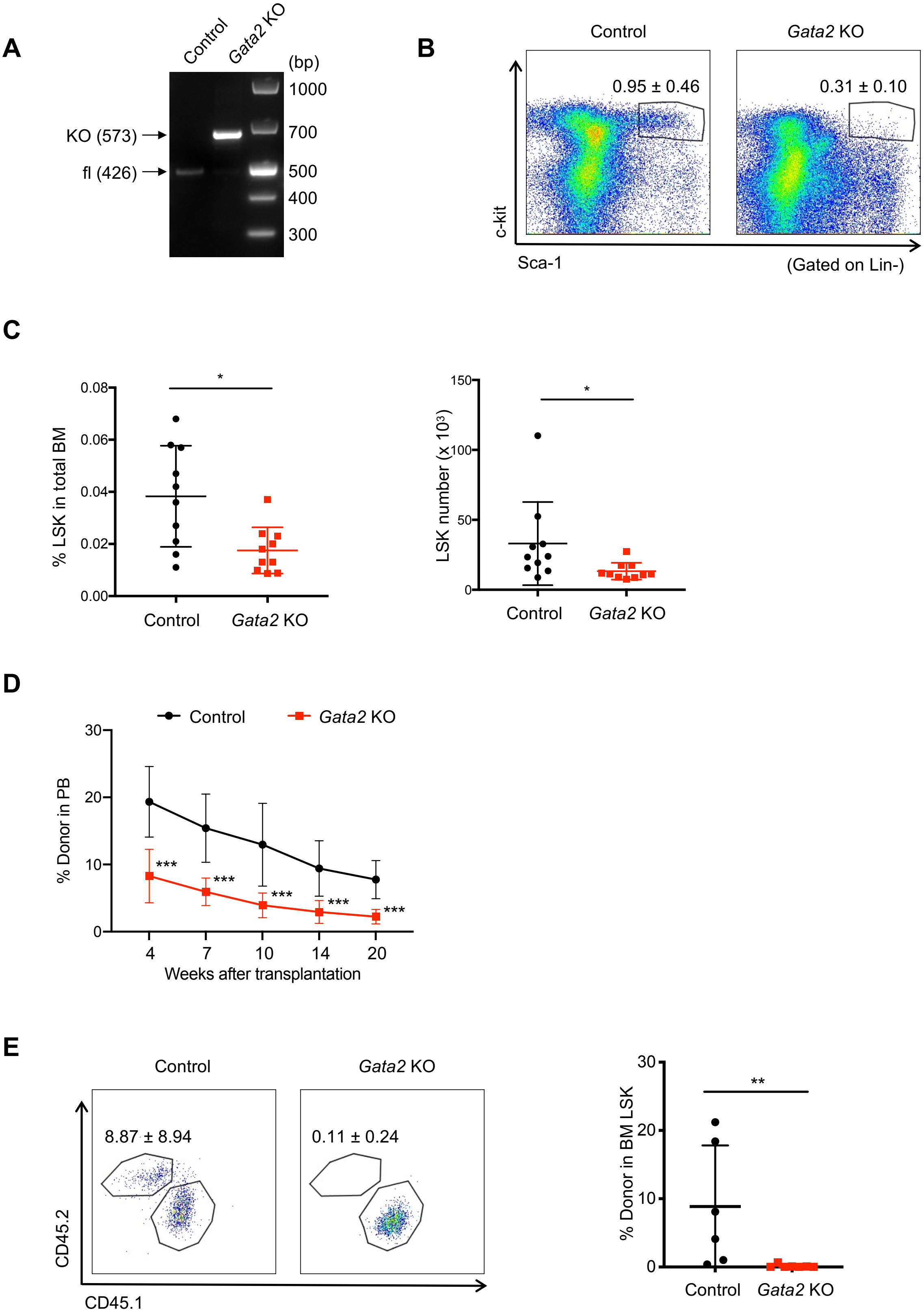

A
Control
Gata2 KO
(bp)
1000
700
500
400
300
KO (573)
fl (426)
B
Control
Gata2 KO
0.95 ± 0.46
0.31 ± 0.10
c-kit
Sca-1
(Gated on Lin-)
C
% LSK in total BM
0.00
0.02
0.04
0.06
0.08
*
Control
Gata2 KO
LSK number (x 10³)
0
50
100
150
*
Control
Gata2 KO
D
Control
Gata2 KO
% Donor in PB
0
10
20
30
***
***
***
***
***
4
7
10
14
20
Weeks after transplantation
E
Control
Gata2 KO
8.87 ± 8.94
0.11 ± 0.24
CD45.2
CD45.1
% Donor in BM LSK
0
10
20
30
**
Control
Gata2 KO

Figure S2.

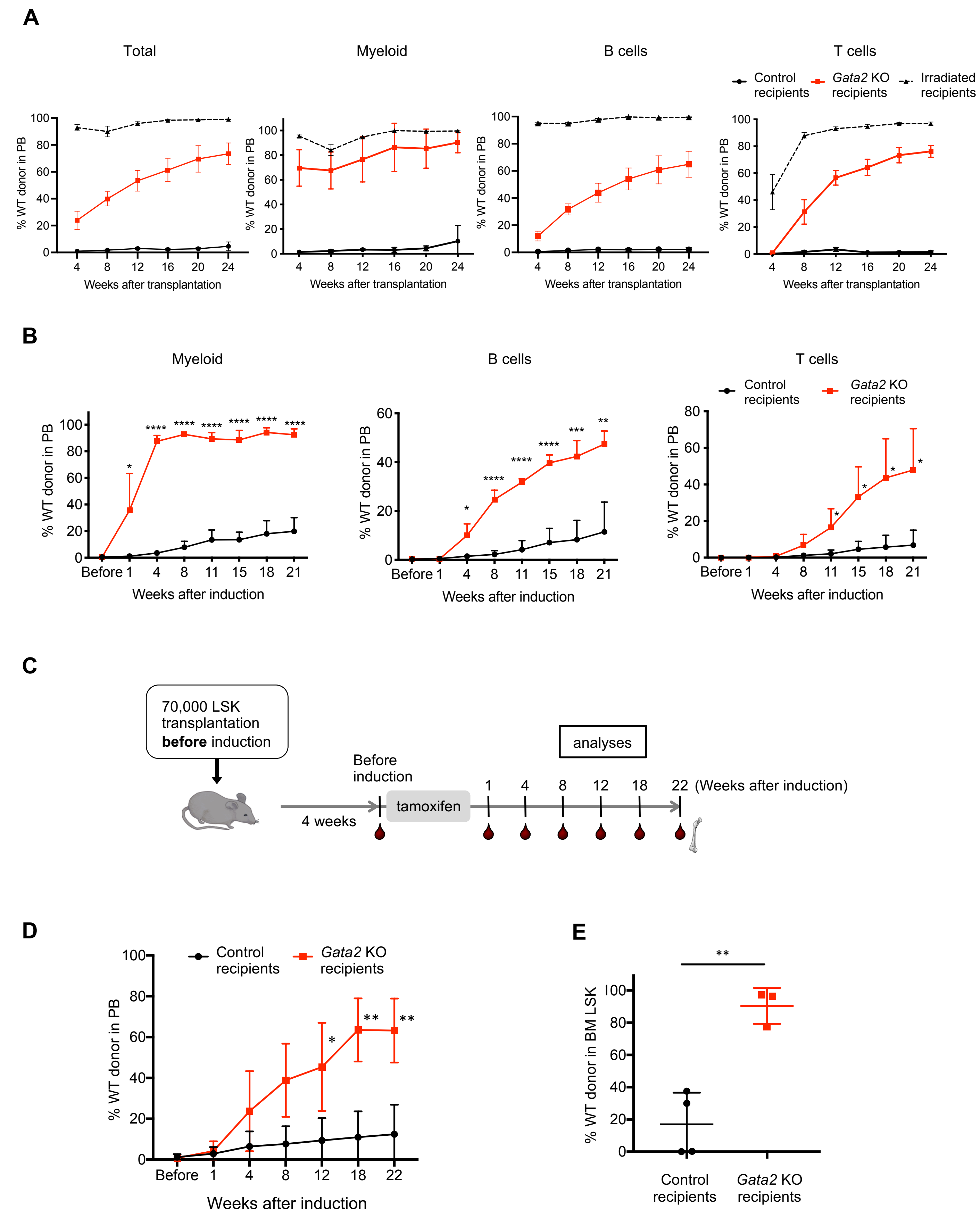
A
Total
Myeloid
B cells
T cells
Control recipients
Gata2 KO recipients
Irradiated recipients
% WT donor in PB
Weeks after transplantation
B
Myeloid
B cells
T cells
Control recipients
Gata2 KO recipients
% WT donor in PB
Weeks after induction
C
70,000 LSK transplantation before induction
4 weeks
Before induction
tamoxifen
analyses
1
4
8
12
18
22 (Weeks after induction)
D
Control recipients
Gata2 KO recipients
% WT donor in PB
Weeks after induction
E
% WT donor in BM LSK
Control recipients
Gata2 KO recipients

Figure S3.

A

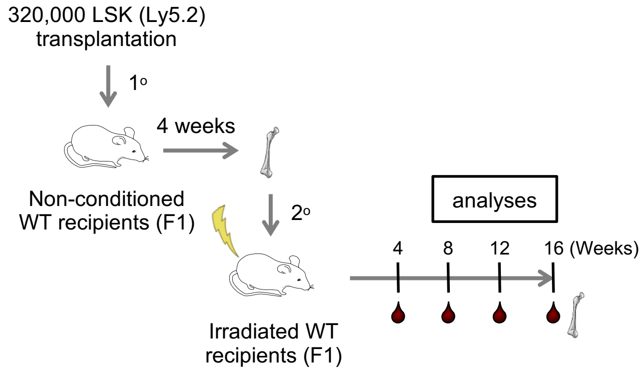

B

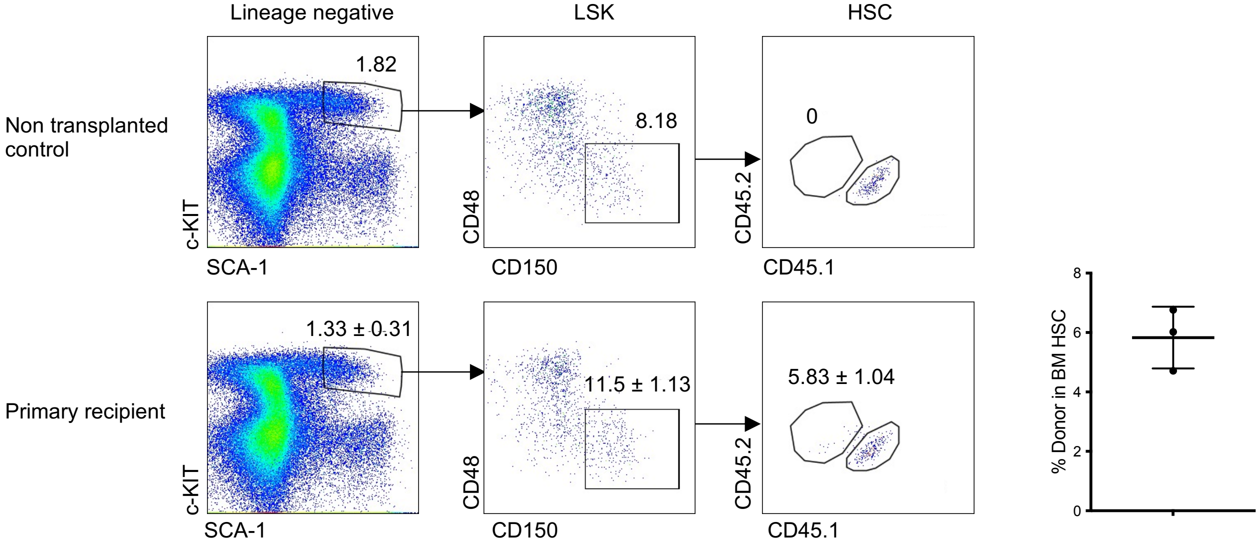

C

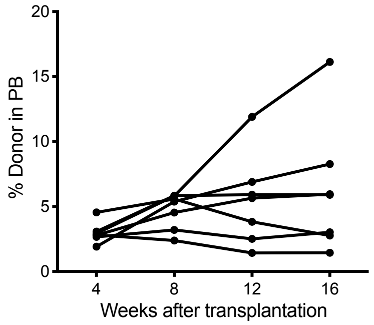

Figure S4.

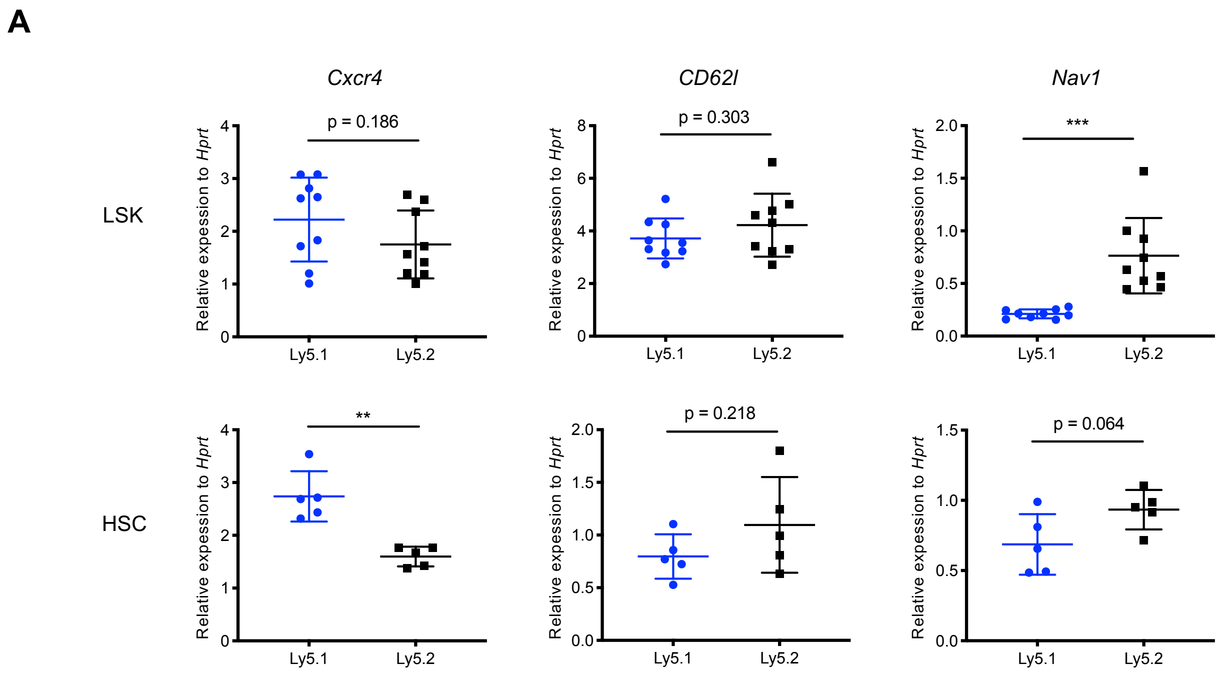
A
Cxcr4
CD62l
Nav1
LSK
HSC
p = 0.186
p = 0.303
***
**
p = 0.218
p = 0.064
Relative expession to Hprt
Ly5.1
Ly5.2

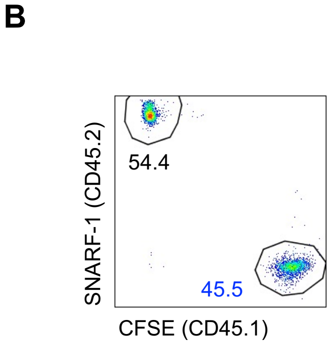
B
SNARF-1 (CD45.2)
CFSE (CD45.1)
54.4
45.5

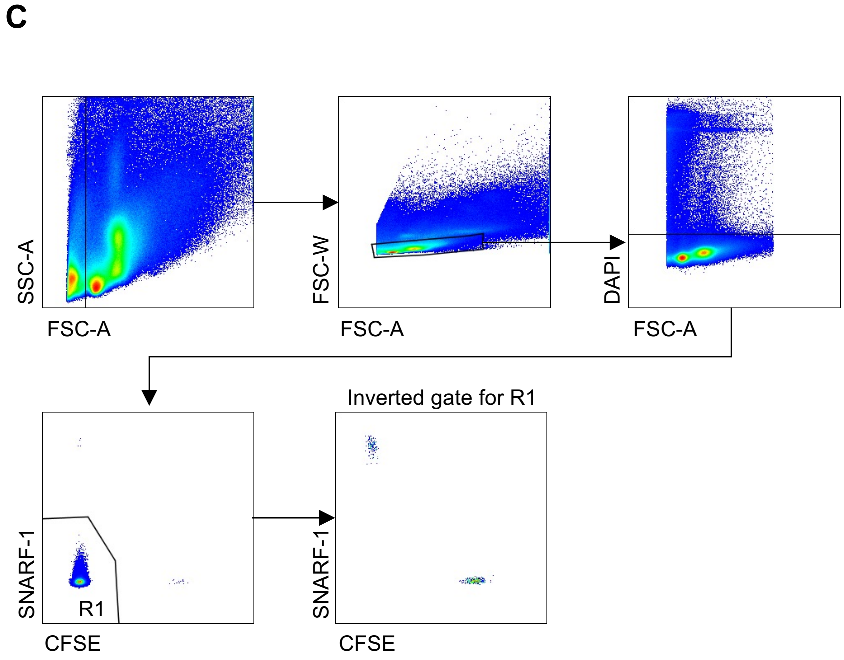
C
SSC-A
FSC-A
FSC-W
DAPI
SNARF-1
CFSE
R1
Inverted gate for R1
