# Supplementary material for: Uncoupling key determinants of hematopoietic stem cell engraftment through cell-specific and temporally controlled recipient conditioning
Source: Stem Cell Reports. 2021 Jun 24;16(7):1705–17. doi: 10.1016/j.stemcr.2021.05.019 (PMC8282468; doi:10.1016/j.stemcr.2021.05.019)
Supplement: Document S2. Article plus supplemental Information [file mmc2.pdf]

Stem Cell Reports
Article
OPEN ACCESS

Uncoupling key determinants of hematopoietic stem cell engraftment through cell-specific and temporally controlled recipient conditioning

Natsumi Miharada,[1] Anna Rydström,[1,3] Justyna Rak,[2,3] and Jonas Larsson[1,*]
[1]Molecular Medicine and Gene Therapy, Lund Stem Cell Center, Lund University, BMC A12, 221 84, Lund, Sweden
[2]Department of Haematology, University of Cambridge, Cambridge, UK
[3]These authors contributed equally
*Correspondence: jonas.larsson@med.lu.se
https://doi.org/10.1016/j.stemcr.2021.05.019

SUMMARY

Hematopoietic stem cells (HSCs) are typically characterized by transplantation into irradiated hosts in a highly perturbed microenvironment. Here, we show that selective and temporally controlled depletion of resident HSCs through genetic deletion of *Gata2* constitutes efficient recipient conditioning for transplantation without irradiation. Strikingly, we achieved robust engraftment of donor HSCs even when delaying *Gata2* deletion until 4 weeks after transplantation, allowing homing and early localization to occur in a completely non-perturbed environment. When HSCs from the congenic strains Ly5.1 and Ly5.2 were competitively transplanted, we found that the more proliferative state of Ly5.2 HSCs was associated with superior long-term engraftment when using conditioning by standard irradiation, while higher CXCR4 expression and a better homing ability of Ly5.1 HSCs strongly favored the outcome in our inducible HSC depletion model. Thus, the mode and timing of recipient conditioning challenges distinct functional features of transplanted HSCs.

INTRODUCTION

The functionality of hematopoietic stem cells (HSCs) can be characterized *in vivo* by transplantation into recipients that lack a functional hematopoietic system (Till and Mc, 1961). Self-renewal and multi-lineage reconstitution capacity can be assessed in transplantation assays, and are the key criteria to define HSCs (Kiel et al., 2005; Osawa et al., 1996; Smith et al., 1991). It is a general notion in the field that the engraftment of donor HSCs is dependent on the availability of niches that normally are occupied by host HSCs (Bhattacharya et al., 2006, 2009; Schofield, 1978; Tomita et al., 1994). Irradiation is therefore used to enhance donor HSC engraftment by depleting resident HSCs and create available niche space (Tomita et al., 1994). However, irradiation destroys not only endogenous HSCs, but also most of the hematopoietic compartment, as well as other cell types in the bone marrow that constitute the niche (Cao et al., 2011; Domen et al., 1998; Dominici et al., 2009). Moreover, irradiation causes a strong inflammatory stress-induced response referred to as a “cytokine storm” (Zhang et al., 2012). Therefore, it is unclear to what degree the functional properties of HSCs assessed in an environment perturbed by irradiation reflect those seen during steady-state hematopoiesis or more physiological challenges (Busch et al., 2015; Busch and Rodewald, 2016; Sun et al., 2014).

While conditioning by irradiation enables robust engraftment and strongly challenges the defining properties of HSCs during acute regeneration, many hematopoietic conditions involving altered HSC function, such as bone marrow failure syndromes or myeloproliferative disorders, develop gradually in a less-challenged context. It would thus be desirable to study HSC regulation under more subtle conditions with limited stress. Furthermore, it is unclear how accurately mechanisms of homing and localization are assessed in irradiated models where the niche has been significantly disrupted (Heazlewood et al., 2014).

Although HSC transplantation into completely non-perturbed recipients can be achieved, this approach is not feasible for most applications since donor engraftment is masked by an overwhelming contribution from endogenous hematopoiesis unless extremely large stem cell numbers are transplanted (Nilsson et al., 1997, 1999; Shimoto et al., 2017). An alternative approach to promote efficient engraftment is to selectively deplete resident HSCs while leaving the more mature hematopoietic cells and surrounding niche components intact. Several studies have shown that employing genetically modified mice with perturbed HSC function as recipients, or specifically depleting host HSCs using antibodies, can facilitate engraftment of transplanted HSCs (Czechowicz et al., 2007; Migliaccio et al., 1999; Wang et al., 2009; Waskow et al., 2009). In such models, hematopoietic reconstitution can be assessed without many of the confounding factors triggered by lethal irradiation. Yet, this context of selective HSC depletion before transplantation is also associated with an acute and heavy demand on the transplanted HSCs, which is quite different from steady-state hematopoiesis. This was demonstrated in a recent study showing that the clonal contribution of transplanted HSCs toward lineage commitment is strikingly different between HSC-depleted and unconditioned recipients (Lu et al., 2019).

Here, we reasoned that a model with selective, as well as temporally controlled depletion of host HSCs would provide

This is an open access article under the CC BY license (http://creativecommons.org/licenses/by/4.0/).

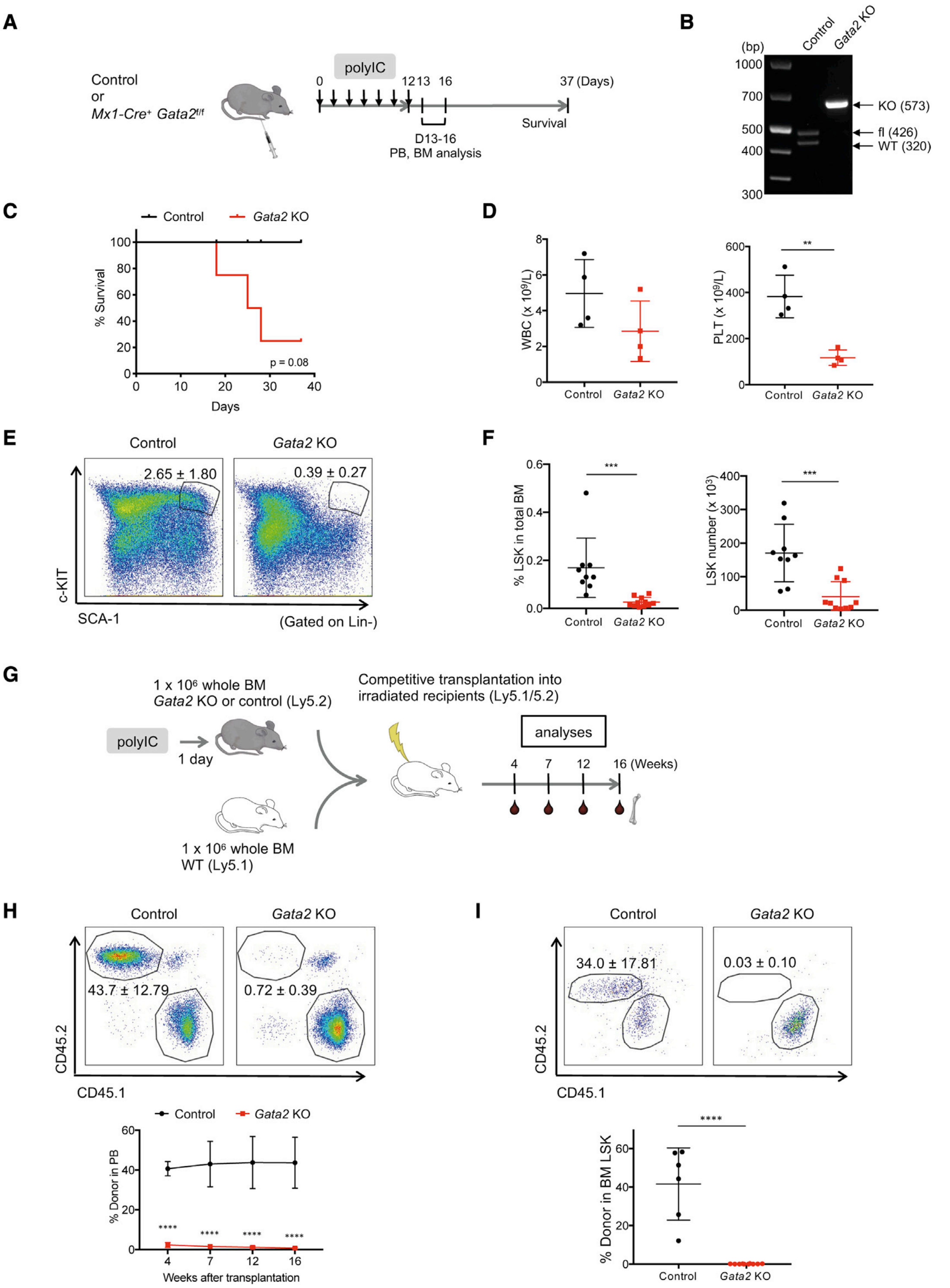

Figure 1. Conditional *Gata2* deletion depletes phenotypic and functional HSCs

(A) *Mx1*-Cre$^+$ *Gata2*$^{fl/fl}$ and control (*Mx1*-Cre$^-$ *Gata2*$^{fl/fl}$ or *Mx1*-Cre$^-$ *Gata2*$^{fl/+}$) mice received seven injections of poly(IC) on alternate days and survival rate was followed until 2 weeks after the last injection. PB and BM were analyzed at 1–4 days after the last poly(IC) injection.
(B) Genomic PCR results with unfractionated BM from *Mx1*-Cre$^+$ *Gata2*$^{fl/fl}$ or control (*Mx1*-Cre$^-$ *Gata2*$^{fl/+}$) mice administered with poly(IC) and harvested at 1 week after the last injection.

(legend continued on next page)

new opportunities for the assessment of HSC dynamics and engraftment after transplantation. We hypothesized that such a model would allow for robust measurements of engraftment properties under completely non-perturbed conditions by depleting resident HSCs not before, but after transplantation. To this end, we established a model where HSCs can be selectively depleted by conditional deletion of *Gata2* (Haugas et al., 2010). GATA2 is a transcription factor that was identified as an essential regulator of HSC generation and survival during embryogenesis (de Pater et al., 2013; Tsai et al., 1994), with an equally critical and dose-dependent role in regulation of adult hematopoiesis (Li et al., 2016; Lim et al., 2012; Ling et al., 2004; Menendez-Gonzalez et al., 2019; Rodrigues, 2005).

Upon induced genetic deletion of *Gata2* in adult mice, we observed rapid and near-complete depletion of HSCs in agreement with previous studies where the cell-intrinsic role of GATA2 has been characterized in conditional knockout (KO) mice (Li et al., 2016; Menendez-Gonzalez et al., 2019). We further demonstrate that this depletion of HSCs allows efficient engraftment of transplanted cells without irradiation. Remarkably, when HSCs from different genetic backgrounds were transplanted in a competitive manner to these mice, we observed fundamentally different outcomes compared with conditioning by irradiation, which further depended on timing of *Gata2* deletion and whether recipient HSCs were depleted before or after transplantation. Specifically, we found that our model enables robust detection of HSCs that have been transplanted into a fully intact microenvironment, and is uniquely suited to assess properties affecting homing and early localization that otherwise are masked when using irradiated recipients.

RESULTS

Induced Cre/*loxP* deletion of *Gata2* by poly(IC) rapidly and selectively depletes the HSC pool in *Mx1*-Cre *Gata2*$^{fl/fl}$ mice

To selectively deplete endogenous HSCs *in vivo*, we used genetically engineered mice bearing conditional alleles of *Gata2* (*Gata2*$^{fl/fl}$ mice) (Haugas et al., 2010). We bred *Gata2*$^{fl/fl}$ mice with *Mx1*-Cre transgenic mice to generate an inducible KO mouse model. Upon poly(IC) administration, *Mx1*-Cre is known to efficiently induce gene deletion in all subsets of hematopoietic stem and progenitor cells (HSPCs) (Kühn et al., 1995). To assess the impact of *Gata2* deletion on hematopoiesis, we administered poly(IC) every second day for 12 days to achieve efficient gene deletion (Figures 1A and 1B). One week after the last poly(IC) injection, the mice started to succumb (Figure 1C) and prior to this we observed a decrease of platelet and white blood cell counts in the peripheral blood (PB) (Figure 1D). Importantly, the phenotypic HSPC compartment (Lineage$^-$ SCA-1$^+$ c-KIT$^+$, LSK) in the bone marrow was markedly reduced (Figures 1E and 1F). To more directly assess the functional impact of *Gata2* deletion on HSCs, we competitively transplanted unfractionated bone marrow (BM) (Ly5.2) from poly(IC)-treated *Mx1*-Cre *Gata2*$^{fl/fl}$ mice or control mice against identical numbers of wild-type (WT) BM (Ly5.1) cells into irradiated recipients (Figure 1G). We detected almost no contribution of *Gata2* KO cells in the PB (Figure 1H) and BM (Figure 1I) of the recipients, while control cells showed a similar level of reconstitution as the WT competitor cells. Altogether, these results demonstrate a rapid and selective depletion of resident HSCs through poly(IC)-induced *Gata2* deletion, consistent with previous reports where *Gata2* has been depleted in adult mice (Li et al., 2016; Menendez-Gonzalez et al., 2019).

Gata2 deletion can serve as a pre-conditioning regimen for transplant recipients and enables efficient HSC engraftment

It has previously been shown that selective HSC depletion can substitute for irradiation as a conditioning regimen for BM transplantation (Czechowicz et al., 2007; Migliaccio et al., 1999; Wang et al., 2009; Waskow et al., 2009). We therefore asked whether the HSC depletion seen in our inducible *Gata2* model would allow for engraftment of transplanted HSPCs and thus we used the inducible *Gata2* KO mice as recipients for transplantation. We first

(C) Kaplan-Meier curve depicting the survival of mice treated with poly(IC) (n = 4).
(D) White blood cell counts and platelet counts analyzed 1 day after the last poly(IC) injection (n = 4).
(E) Representative FACS plots showing the LSK compartment at 1 day after the last poly(IC) injection.
(F) Frequency (left) and cell number (right) of LSK from *Gata2* KO and control mice at 1–4 days after the last poly(IC) injection. Data are pooled from three independent experiments (control, n = 9; *Gata2* KO, n = 11).
(G) 1×10^6 of whole BM cells (Ly5.2) from *Mx1*-Cre$^+$ *Gata2*$^{fl/fl}$ or control (*Mx1*-Cre$^-$ *Gata2*$^{fl/+}$) mice administered with poly(IC) were transplanted together with equal numbers of whole BM cells from WT (Ly5.1) into irradiated recipients in a competitive manner (three to four donors per genotype and two to three recipients per donor).
(H and I) Frequency of Ly5.2 cells in PB (H) and BM (I) after transplantation. Representative FACS plots showing the contribution from each donor at 16 weeks after transplantation (control, n = 6; *Gata2* KO, n = 10). n represents independent biological replicates, except in (H and I) where n represents technical replicates (recipient mice) from three to four independent biological repeats (donor mice).
See also Figure S1.

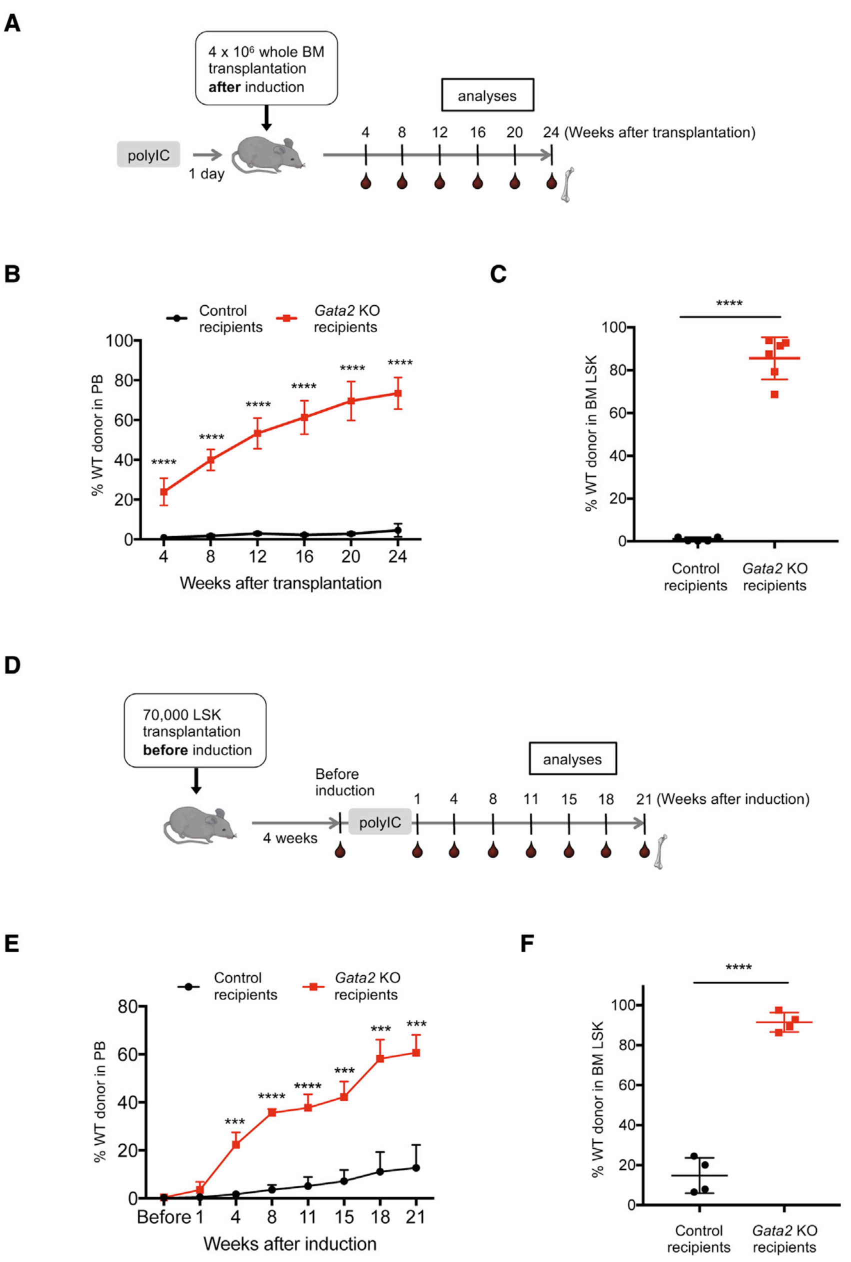

Figure 2. *Gata2* deletion enables efficient engraftment of transplanted HSCs

(A) *Mx1*-Cre$^+$ *Gata2*$^{fl/fl}$ and control (*Mx1*-Cre$^-$ *Gata2*$^{fl/fl}$ or *Mx1*-Cre$^-$ *Gata2*$^{fl/+}$) mice were treated with poly(IC) before the transplantation. One day after the last injection, 4×10^6 whole BM cells were transplanted. Donor contribution was analyzed for 24 weeks after the transplantation.

(B) Frequency of donor-derived cells in PB after the transplantation (n = 5–6).

(C) Frequency of donor-derived cells in BM LSK at 24 weeks after the transplantation (n = 5–6).

(D) *Mx1*-Cre$^+$ *Gata2*$^{fl/fl}$ mice were transplanted with 70,000 LSK cells without any conditioning. Four weeks after the transplantation, half of the recipients were treated with poly(IC). Donor contribution was monitored for up to 21 week after the last poly(IC) injection.

(E) Frequency of donor in PB was analyzed before poly(IC) injection (4 weeks after the transplantation) and followed up to 21 weeks after the last poly(IC) injection (n = 4).

(F) Frequency of donor in BM LSK at 21 weeks after the last poly(IC) injection (n = 4). n represents independent biological replicates. See also Figures S2 and S3.

administered poly(IC) to the *Mx1*-Cre *Gata2* and control mice. One day after the last injection, unfractionated WT BM cells were transplanted into the poly(IC)-treated mice (Figure 2A). We saw a gradual increase in donor engraftment in PB, and a robust reconstitution of the HSPC compartment in the BM of the *Gata2*-deficient recipients, while control recipients showed barely detectable levels of donor cells (Figures 2B and 2C). Notably, the kinetics of the overall reconstitution levels showed a somewhat delayed pattern compared with standard conditioning by lethal irradiation (Figures 2B and S2A). The delayed reconstitution kinetics were especially prominent within the lymphoid compartment, while the reconstitution levels of myeloid cells rapidly reached a plateau, as seen in irradiated recipients (Figure S2A). Since our HSC depletion model, unlike irradiation, spares progenitors and mature cells, it takes longer to replace the more long-lived lymphoid compartment (B and T cells) in the recipients, while the short-lived myeloid cells are rapidly replenished by the donor HSPCs.

Taken together, our findings demonstrate that induced deletion of *Gata2* can serve as a conditioning regimen for

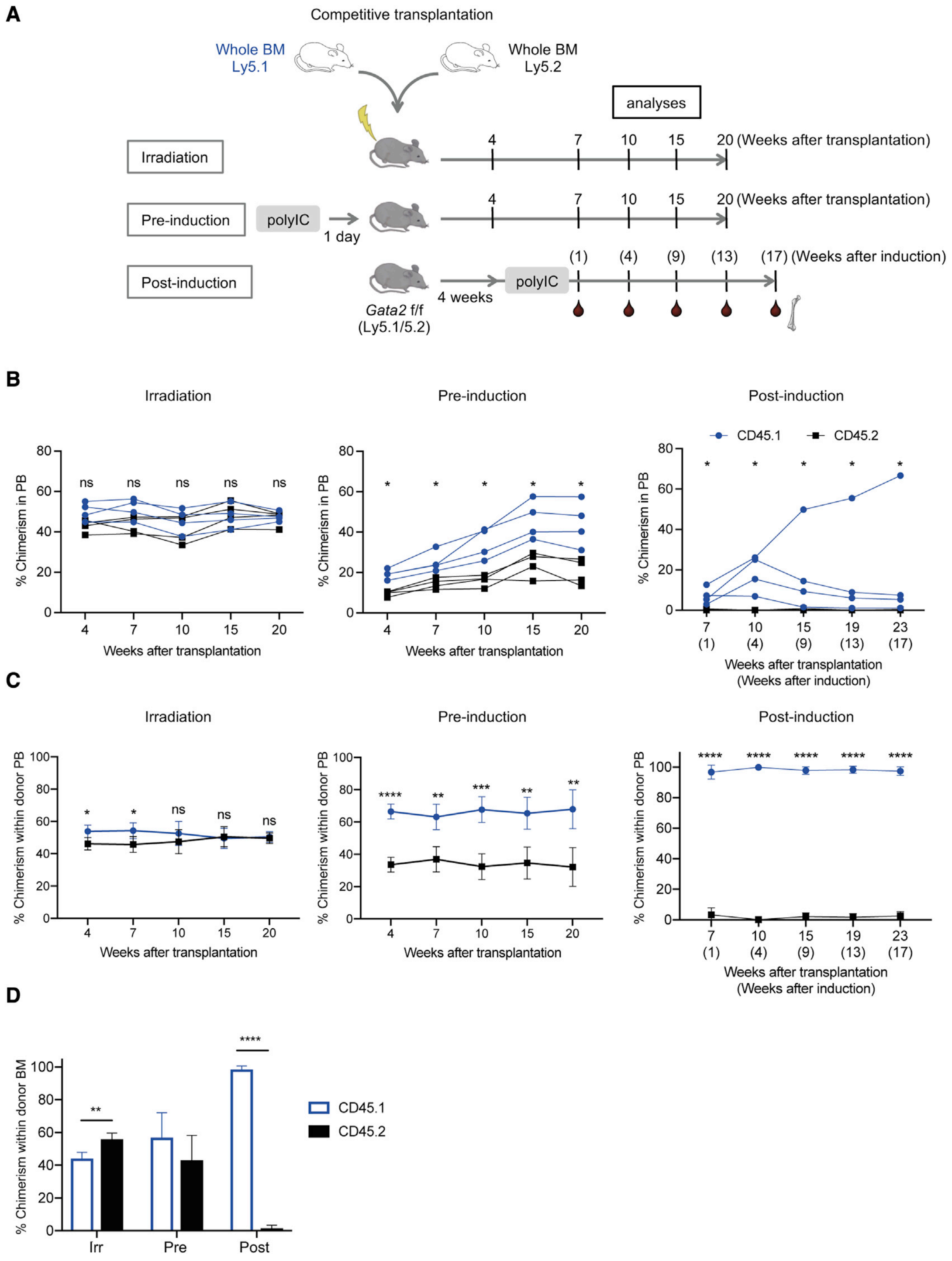

Figure 3. Engraftment kinetics depend on the mode of recipient conditioning

(A) Equal number of unfractionated BM cells from Ly5.1 and Ly5.2 mice were transplanted into the recipients at three different conditioning regimens. For irradiated and pre-induction setting, one million cells each from Ly5.1 and Ly5.2 donors were co-transplanted. For post-induction setting, ten million cells from each strain were co-transplanted.

(legend continued on next page)

transplant recipients and enables efficient engraftment of transplanted HSCs without irradiation.

HSPCs transplanted to non-irradiated, non-induced *Mx1*-Cre *Gata2*$^{fl/fl}$ mice show robust engraftment upon subsequently induced *Gata2* deletion

Next, we explored an alternative transplantation approach where donor HSPCs were transplanted and allowed to initially engraft in a completely non-perturbed environment. Non-induced *Mx1*-Cre *Gata2* mice were intravenously injected with 70,000 WT LSK cells, and 4 weeks later host HSCs were depleted by poly(IC) administration (Figure 2D). We hypothesized that this selective host HSC depletion through loss of *Gata2* would gradually reduce the host-derived hematopoiesis and promote a selective expansion of hematopoiesis from the previously transplanted donor cells. As expected, almost no donor cells were detected in the PB of the recipients before poly(IC) induction (Figure 2E). Strikingly, however, donor reconstitution gradually but substantially increased following poly(IC) treatment and was subsequently sustained in the blood long term (Figure 2E). Similar to the other model, myeloid reconstitution was rapid and lymphoid reconstitution was more delayed (Figure S2B). We further detected high levels of donor engraftment in the most primitive BM populations (Figure 2F). Control *Mx1*-Cre *Gata2*$^{fl/fl}$ mice that had not been administered poly(IC) showed detectable, albeit very low levels of engraftment, which is likely due to leakiness of the *Mx1*-Cre system over time with spontaneous *loxP* recombination of *Gata2* (Velasco-Hernandez et al., 2016). These results demonstrate that engraftment of HSCs in non-conditioned hosts can be robustly detected following subsequent depletion of the endogenous HSC pool.

Interferon alpha induced by poly(IC) has been shown to promote the HSC proliferation (Essers et al., 2009). To exclude the possibility that the poly(IC)-mediated interferon alpha response would have significantly impacted the results, we corroborated our findings using an independent model of *Gata2* deletion. *Gata2*$^{fl/fl}$ mice were crossed with the estrogen responsive *Rosa26*-Cre-ER$^{T2/+}$ (ER-Cre) mice (Ventura et al., 2007) to generate a model enabling *Gata2* deletion upon tamoxifen treatment (Figure S1A). Similar to the *Mx1*-Cre model, we observed the rapid depletion of HSPCs within days after tamoxifen induction as measured by both FACS and competitive transplantation (Figures S1B–S1E). Moreover, we could replicate the finding of efficient repopulation from HSCs that previously had been engrafted in completely non-conditioned animals, by subsequently deleting *Gata2* in the recipients (Figures S2C–S2E). Transplanted HSCs must thus be able to persist in non-conditioned recipients for at least 4 weeks with preserved potential, and we next asked whether they were localized within the BM environment. BM from non-conditioned WT recipient mice was harvested 4 weeks following transplantation of LSK cells (Figure S3A). We found clearly detectable levels of donor HSCs by FACS (Figure S3B), and secondary transplantation showed that they had robust long-term engraftment potential (Figure S3C). This shows that transplanted HSCs localize to the BM in non-conditioned recipients and that they subsequently can be recruited upon demand.

Altogether, our findings demonstrate that the deletion of resident HSCs through endogenous *Gata2* deletion provides a versatile model for engraftment studies in non-irradiated animals, with possibility of recipient conditioning either before or after transplantation.

The engraftment kinetics of transplanted HSPCs from the Ly5.1 and Ly5.2 congenic strains differ dramatically depending on the mode of recipient conditioning

To explore the impact of the different modes of transplantation conditioning on the functional readout of HSPCs, we employed the Ly5.1/5.2 competitive transplantation assay in our model. This assay is based on variants of the Ly5 antigen, which is expressed on all leukocytes. The Black6 Ly5.1 strain is congenic with the C57/Black6 Ly5.2 strain and differs only at the genomic region that covers the Ly5 locus. Yet, it has been observed that this minor genetic discrepancy has functional consequences at the HSC levels that the Ly5.2 strain shows a subtle competitive advantage over the Ly5.1 strain in transplantation assays (Mercier et al., 2016; Waterstrat et al., 2010). We therefore asked whether these subtle differences in HSC function between the two congenic strains would read out differently depending on the mode of recipient conditioning. Equal numbers of whole BM cells from Ly5.1 and Ly5.2 mice were mixed and injected into lethally irradiated recipients, as well as into our *Gata2* recipient model, using either *Gata2* deletion before transplantation (pre-induction) or 4 weeks after transplantation (post-induction) as described above. To ensure that the Ly5.1 and Ly5.2 cells would be accepted equally by the host, we used an Ly5.1/5.2 F1 background of the *Gata2* mice as recipients (Figure 3A). As expected, the irradiated recipients showed rapid

(B) Frequency of Ly5.1 and Ly5.2 cells in PB from individual recipients after the transplantation (n = 4).
(C) Relative contribution of Ly5.1 and Ly5.2 cells within the donor-derived population in PB after the transplantation (n = 4).
(D) Relative contribution of Ly5.1 and Ly5.2 cells within donor-derived population in BM at 20 weeks after the transplantation. For post-treated group, 17 weeks after the last poly(IC) injection (n = 4). n represents independent biological replicates.

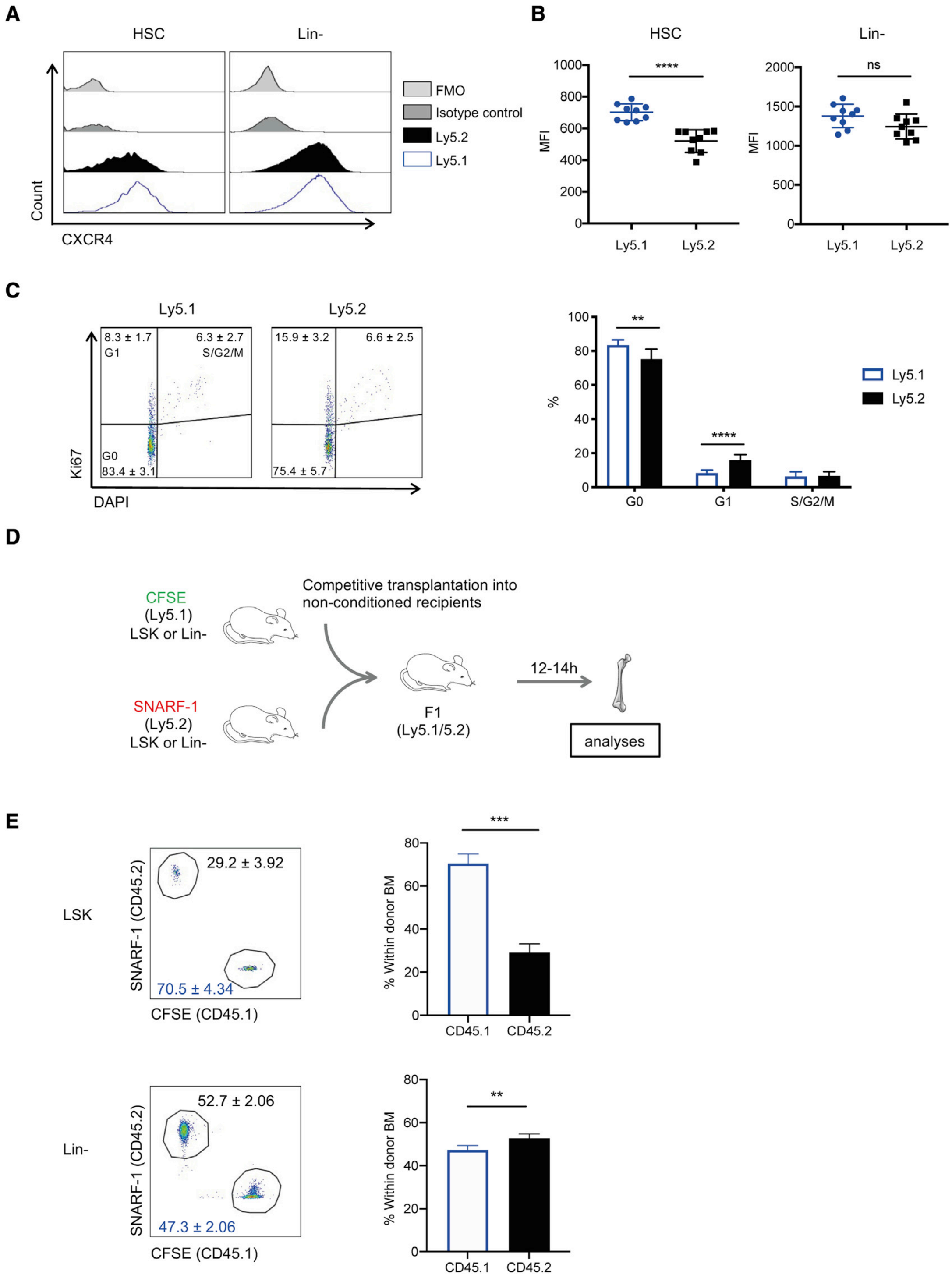

Figure 4. Advantage in homing is associated with higher engraftment potential in non-irradiated recipients

(A) Representative histogram plots showing cell surface expression levels of CXCR4 protein in HSC (left) and Lin (right) populations of Ly5.1 and Ly5.2 mice.

(B) Mean fluorescence intensity (MFI) of CXCR4 protein in HSC (left) and Lin (right) populations of Ly5.1 and Ly5.2 mice. Data were pooled from three independent experiments (n = 9).

(legend continued on next page)

and efficient donor reconstitution, while engraftment in the *Gata2* models was more delayed and with overall engraftment levels being lower in the post-induction setting (Figure 3B). To more directly assess the competition between transplanted HSCs from the Ly5.1 and Ly5.2 strains, we determined the relative contribution of Ly5.1 and Ly5.2 cells within the overall donor-derived population excluding the contribution from the Ly5.1/5.2 F1-recipient mice (Figure 3C). In the irradiated recipients, Ly5.1 cells showed a minor advantage over Ly5.2 at earlier time points, and a tendency to decrease at later time points with the anticipated advantage of Ly5.2 seen in the long-term BM analysis (Figures 3C and 3D). In the pre-induction model, however, the Ly5.1 cells showed an advantage over Ly5.2 throughout the transplantation assay (Figures 3B and 3C). Even more strikingly, the Ly5.1 cells almost completely outcompeted their Ly5.2 counterparts in the post-induction model. The relative contribution from Ly5.1 cells in the PB exceeded 90% already 1 week after poly(IC) administration (Figure 3C) and this was sustained long term in the BM (Figure 3D). Thus, our findings show that the *in vivo* engraftment kinetics of transplanted HSCs from the Ly5.1 and Ly5.2 congenic strains differ dramatically depending on the mode of conditioning used for the recipient mice.

HSPCs from the Ly5.1 and Ly5.2 strains show differential levels of CXCR4 expression and differ in their homing ability to the BM

We next wanted to determine the basis for the different outcomes in our transplantation models and the remarkably higher engraftment of 5.1 HSPCs seen in the post-induction model. From genetic and molecular analysis of the Ly5.1 and Ly5.2 strains, it has been shown previously that there is a congenic interval in the genome containing around 300 genes, some of which may be differentially expressed (Chisolm et al., 2019; Waterstrat et al., 2010). Among these are several adhesion- and migration-related molecules that potentially could influence the engraftment process, including cadherin 19 (*Cdh19*), chemokine receptor 4 (*Cxcr4*), engrailed 1 (*En1*), neuron navigator 1 (*Nav1*), Slit-Robo GTPase-activating protein 2 (*Srgap2*), and L-Selectin (*Sell/CD62*l). Based on data from available databases (Gene Expression Commons https://gexc.riken.jp/, Blood Spot http://servers.binf.ku.dk/bloodspot/), we selected those that were either broadly expressed in hematopoietic cells (*Cxcr4*, *CD62*l), enriched in progenitors (*Nav1*), or that lacked expression data (*Cdh19*). We confirmed expression of these genes in both LSK cells and HSCs (CD150^{+}CD48^{-} LSK), except Cdh19 which could not be detected (Figure S4A). Among these expressed genes, only *Cxcr4* showed higher expression in Ly5.1 compared with Ly5.2 cells, and this difference was specific to purified HSCs while less prominent in the bulk LSK population. Moreover, CXCR4 is known to have a critical role in the retention of HSPCs in the BM (Broxmeyer et al., 2005; Foudi et al., 2006). This prompted us to further examine the potential relevance of CXCR4 expression levels for the differential HSPC function between the Ly5.1 and Ly5.2 strains. We saw significantly higher expression of CXCR4 also at the protein level in Ly5.1 HSCs (CD150^{+}CD48^{-} LSK) compared with Ly5.2 counterparts (Figures 4A and 4B). This difference was specific to HSCs and not detected within the bulk of lineage-negative (Lin^{-}) cells in agreement with our gene expression results showing differential expression mainly within the HSC compartment. CXCR4 has been shown to regulate the maintenance of the quiescent HSC pool and its loss is associated with increased cycling of HSCs (Nie et al., 2008; Sugiyama et al., 2006). Accordingly, cell-cycle analysis of Ly5.1 and Ly5.2 HSCs revealed that a significantly higher proportion of Ly5.1 HSCs resided in the G0 phase, consistent with their higher CXCR4 expression, while the G1 phase had a higher proportion of Ly5.2 HSCs (Figure 4C).

Next, we asked if the level of CXCR4 expression in HSC affects their homing ability to the BM and whether this could explain the higher engraftment potential of Ly5.1 HSCs in the post-induction model. We assessed homing of Ly5.1 and Ly5.2 HSPCs in a competitive manner by labeling LSK cells from Ly5.1 and Ly5.2 mice with either the fluorescent dyes 5- (and -6-) carboxyfluorescein diacetate succinimidyl ester (CFSE) or seminaphtorhodafluor-1 carboxylic acid acetate succinimidyl ester (SNARF-1) (Figure S4B), followed by competitive transplantation into Ly5.1/5.2 F1 recipients without irradiation (Figure 4D). Twelve hours after the transplantation, we observed a significantly higher recovery of Ly5.1 cells in the BM of the recipients (Figures 4E and S4C). No difference was detected when lineage-negative cells were analyzed in the same manner, consistent with the CXCR4 expression

(C) Cell-cycle analysis showing the frequency of G0, G1, and S/G2/M phases in HSCs from Ly5.1 and Ly5.2 mice. Data are pooled from three independent experiments (n = 9).
(D) Equal numbers of CFSE- or SNARF-1-labeled cells were co-transplanted into the recipients without irradiation. After 12–14 h, the cells homed to the BM were isolated and analyzed using FACS.
(E) Representative FACS plots (left) and relative frequency (right) of donor-derived CFSE- and SNARF-1-positive cells homed to the BM after 12–14 h (LSK, n = 3; Lin^{-}, n = 6). n represents independent biological replicates.
See also Figure S4.

data, where the difference in expression was specific to HSCs and not seen in the bulk population. These findings show superior homing ability of Ly5.1 HSPCs, which correlates with a higher expression of CXCR4. Taken together, our results suggest that the superior engraftment potential of Ly5.1 HSCs seen in our *Gata2* model can be explained, at least in part, by their higher CXCR4 expression, which may provide an advantage during the early engraftment process. Moreover, the findings suggest that homing and early engraftment are less dependent on CXCR4 in the lethally irradiated setting. Our temporally controlled model of HSC depletion provides a highly sensitive approach to assess regulators of homing and early localization in functionally defined HSCs.

DISCUSSION

Transplantation into irradiated animals has been extensively used as a gold standard method to assess the functionality of HSCs *in vivo*, allowing assessment of multi-lineage reconstitution and self-renewal. In such models, transplanted HSCs can engraft efficiently and are forced to actively contribute the hematopoiesis to replenish the hematopoietic system disrupted by irradiation. However, irradiation severely damages cellular components of the microenvironment (Cao et al., 2011; Domen et al., 1998; Dominici et al., 2009), and thus transplantation in this setting limits assessment of the interaction between transplanted HSCs and the microenvironment. The temporally controlled and selective HSC depletion model presented in this study is a versatile tool for studies of HSC engraftment kinetics in a preserved microenvironment. Our findings further highlight the complexity of the engraftment process and the importance of recipient context when assessing HSC function using transplantation.

Successful engraftment of intravenously transplanted HSCs in the BM requires several steps. Homing is the first step whereby transplanted HSCs are chemoattracted to the BM, interact with the BM vasculature, and migrate into the extravascular space. Subsequently they localize to specific niches and can then either enter a dormant state or be recruited into active hematopoiesis (Heazlewood et al., 2014). Studies in KO mice have shown CXCR4 expression of HSCs and CXCL12 expression in the BM to be critical in the homing process through a chemoattraction mechanism (Foudi et al., 2006). Recent studies have further demonstrated mechanistic insights on the role of CXCR4 in the homing of HSPCs in irradiated recipients, and that modulation of the interaction between CXCR4 and its ligand CXCL12 is a useful approach to facilitate homing (Capitano et al., 2015; Guo et al., 2017; Huang et al., 2018). Our findings from co-transplanting Ly5.1 and Ly5.2 cells, indicate that even minor differences in CXCR4 expression affect the homing ability in non-conditioned hosts. The competition for the limited available niche space in an unperturbed environment may be more dependent on CXCR4 expression, and possibly other adhesion and migration mechanisms, compared with an irradiated or HSC-depleted microenvironment. As such, engraftment studies in irradiated animals may not reflect the normal physiology of homing and localization of HSCs in an optimal way. Homing assays using bona fide HSCs have typically been challenging to perform as it is difficult to recover sufficient numbers of cells for meaningful measurements and due to the dependence of these assays on a phenotypic definition of HSCs. The ability of our model to first allow transplanted HSCs to localize within the BM and subsequently recruit them into active hematopoiesis by depleting resident HSCs provides a highly sensitive measure of homing/localization coupled with a functional HSC definition as a final readout.

It is striking that the Ly5.1 and Ly5.2 congenic strains that differ only in a limited genetic interval (300 genes) show two distinct differences in HSC function. One being the previously demonstrated competitive advantage of Ly5.2 HSCs in standard long-term reconstitution assays (Mercier et al., 2016; Waterstrat et al., 2010), and the other the relative homing advantage of the Ly5.1 counterparts, as demonstrated in this study. Our findings indicate that at least the latter effect is functionally associated with higher CXCR4 expression seen in Ly5.1 HSCs, and has a dramatic impact on long-term engraftment in our post-induction model. The initially non-pertubed microenvironment in this model allows overall lower levels of engraftment, but is highly sensitized to detect differences in homing and early localization. By contrast, our findings indicate that such differences are largely masked in irradiated recipients and that the proliferative capacity of HSCs is a stronger determinant of long-term reconstitution in that context. Whether the competitive advantage of the Ly5.2 HSCs in the irradiated setting also relates to CXCR4 expression or another gene within the congenic interval remains to be investigated. Yet, loss of CXCR4 expression has been associated with increased proliferation of HSCs that does not compromise their long-term maintenance (Nie et al., 2008). It is thus possible that the relatively lower CXCR4 expression of Ly5.2 HSCs contributes to a more active cell-cycle status and thereby promotes their contribution to hematopoiesis in the long term.

A general notion in the field is that HSC niches have to be emptied of resident stem cells to allow efficient engraftment of transplanted cells. Although our findings are generally compatible with this notion, it may be overly simplistic. We show here that the inability to detect robust engraftment levels using non-conditioned recipients, is at

least to some degree due to an overwhelming competitive load from the host compartment. Indeed, when this load is reduced upon *Gata2* deletion 4 weeks after transplantation, it is clear that previously non-detectable donor HSCs are recruited and significantly contribute to hematopoiesis. There is thus substantial engraftment of donor HSCs in the absence of any conditioning and niche clearance. This is supported by studies showing that extremely large doses of donor HSCs can compensate for the relative inability to engraft in non-conditioned hosts and that direct competition from host HSCs is a key limiting factor for engraftment (Colvin et al., 2004; Shimoto et al., 2017; Westerhuis et al., 2011). Yet, the fact that we observed overall lower engraftment levels in the post-induction setting suggests that niche availability at the time of transplantation is still a strong determinant of engraftment efficiency.

We have shown that distinct functional properties of transplanted HSCs involving either homing/localization or proliferation are differentially challenged depending on the conditioning regimen. Our findings illustrate how even discrete genetic differences, as seen between the congenic strains Ly5.1 and Ly5.2, can result in fundamentally different outcomes. Importantly, as most of the genetic determinants of HSC function described to date have been characterized using transplantation assays in irradiated recipients, it is very likely that key features have been missed, particularly those involving homing/localization. Our model provides a useful approach to dissect these different functions, through adjustable timing of endogenous HSC depletion with minimal impact on the microenvironment. Although an inducible KO is a somewhat artificial system that does not reflect normal physiological hematopoiesis, and may not mirror the human setting, there are several unique aspects of our model that we believe will be useful to other scientists in the field. Particularly the post-induction setting that allows the assessment of donor engraftment in a completely intact environment is a specific advantage of our model that cannot be achieved by other reported strategies to facilitate donor engraftment without irradiation through HSPC-specific depletion. Niche interactions and mechanisms of HSC competition for niche space may differ fundamentally depending on niche availability and the degree of niche perturbation. In this context, it may be of particular interest to study malignant hematopoiesis in our model to assess the dynamics and interactions of malignant cells with the BM microenvironment, and their competition with healthy hematopoiesis (Akinduro et al., 2018). Yet, it should be acknowledged that one limitation of our model is that it requires access to the conditional KO strain and therefore takes longer to establish and is less available than regular WT recipient mice.

Taken together, we conclude that our model provides better possibilities to carefully assess the interaction between transplanted HSCs and the microenvironment in relation to engraftment kinetics and transplantation outcome, which will have implications for both basic HSC research as well as clinical transplantation. Clinically there is increasing interest in non-genotoxic conditioning, especially for currently emerging gene therapy applications. Better understanding of the determinants of the engraftment process in this setting should inform the optimization of donor cell numbers and conditions required for successful cell therapy procedures without genotoxic conditioning.

EXPERIMENTAL PROCEDURES

Mice

All animals were maintained in the animal facility at the biomedical center, Lund University, and kept in ventilated racks and given autoclaved food and water. All experiments were approved by the Lund University Animal Ethical Committee. Genetically engineered mice bearing conditional alleles of *Gata2* (*Gata2*$^{fl/fl}$ mice) were obtained from Dr. M. Salminen (University of Helsinki, Finland) (Haugas et al., 2010). The mice were backcrossed at least four generations on a C57BL/6 background and bred with *Mx1*-Cre^{+} and Rosa26Cre-ER$^{T2/+}$ transgenic mice to generate an inducible KO mouse model. *Mx1*-Cre^{+} *Gata2*$^{fl/fl}$ and ER-Cre^{+} *Gata2*$^{fl/fl}$ mice were crossed with B6SJL strain to create an inducible KO model on Ly5.1/5.2 F1 background. *Gata2* conditional gene deletion in the *Mx1*-Cre model was achieved by administration of four to seven intraperitoneal injections of poly(IC) every other day (400 μg per dose; Sigma P1530). For the ER-Cre model, mice were treated with 5 consecutive days of tamoxifen (1 mg per dose; Sigma T5648) reconstituted in peanut oil. Cre-mediated *Gata2* deletion was verified by PCR with primers *Gata2*-Fwd (5′-CAGGCCTTTACCTGTTCCAG-3′), *Gata2*-Rev (5′-GGACCGA AACCCCTAAAGAA-3′), and *Gata2*-KO2-Rev (5′-AGCGAGGGCT TAGTAGCTCA-3′) using the following parameters: 95°C for 5 min, followed by 36 cycles of 95°C for 45 s, 60°C for 45 s, 72°C for 2 min, and then 72°C for 10 min. For Ly5.1/5.2 competitive transplantation, Ly5.1 (B6.SJL) and F1 (Ly5.1/5.2; C57BL/6xB6SJL) mice were obtained from in-house breeding at Lund University. Ly5.2 (C57BL/6N) mice were obtained from Janvier.

Cell preparation

PB was collected from the tail vein and kept in Microvette tubes (Sarstedt) for further analysis. Blood parameters were analyzed on Sysmex XE-5000 (Sysmex Europe). For the donor contribution analysis, red blood cells were lysed with ammonium chloride (NH_4Cl; STEMCELL Technologies) and stained with antibodies. BM cells were isolated by crushing tibias, femurs, and iliac bones in a mortar with pestle. For LSK (Lineage^{-} SCA-1^{+} c-KIT^{+}) sorting, BM cells from spine and sternum were also collected. Single-cell suspensions were prepared by filtering through a 40 μm nylon cell strainer (Fisher Scientific). Lin^{-} cells were obtained using a lineage cell depletion kit (Miltenyi Biotec) according to the manufacturer's instructions.

Flow cytometry and cell sorting

c-KIT-positive cells were enriched using a magnetic separation system (MACS) with anti-c-KIT magnetic beads (Miltenyi Biotec). The enriched cells were stained with c-KIT-APC (2B8, BioLegend), SCA-1-PE (E13–161.7, BioLegend), and lineage antibody cocktail (CD3, B220, CD11b, Gr-1, and Ter119; all from BD) conjugated with PE-Cy5. Dead cells were excluded using 7-amino-actinomycin-D staining. For the analysis of CXCR4 protein expression, c-KIT-enriched BM cells were stained with CXCR4-PE (2B11, eBioscience) or Isotype Control (eB149/10H5, eBioscience) together with CD48-FITC (HM48-1, BioLegend), CD150-PE-Cy7 (TC15-12F12.2, BioLegend), c-KIT-APC-eFluor780 (2B8, Fisher Scientific), SCA-1-Brilliant Violet 421 (D7, BioLegend), and lineage antibody cocktail conjugated with PE-Cy5. $CD150^{+}CD48^{-}$ within LSK cells were defined as HSCs (Kiel et al., 2005). Cells were sorted on FACSAria III or FACSAria IIu and analyzed on FACS Fortessa, LSRII, or FACSCanto II (BD). Collected data were analyzed with FlowJo software (Tree Star).

Transplantation assay

For competitive transplantation of *Gata2* model as donor, 1×10^6 unfractionated BM cells from *Mx1*-Cre^{+} *Gata2*$^{fl/fl}$ or control (*Mx1*-Cre^{-} *Gata2*$^{fl/fl}$ or *Mx1*-Cre^{-} *Gata2*$^{fl/+}$) mice (Ly5.2) that received 7 injections of poly(IC) were harvested at 1 day after the last injection and transplanted into lethally irradiated (900 cGy) recipients (Ly5.1/5.2 F1) along with 1×10^6 unfractionated BM cells from WT mice (Ly5.1). For the transplantation into *Gata2* model as recipients at pre-induction setting, *Mx1*-Cre^{+} *Gata2*$^{fl/fl}$ and control (*Mx1*-Cre^{-} *Gata2*$^{fl/fl}$ or *Mx1*-Cre^{-} *Gata2*$^{fl/+}$) mice received 6 injections of poly(IC) and 1 day after the last injection, 4×10^6 unfractionated BM from WT mice (F1) were transplanted. Same cells were transplanted into lethally irradiated control recipients. For post-induction setting, 70,000 LSK cells were transplanted into *Mx1*-Cre^{+} *Gata2*$^{fl/fl}$ mice without any treatment and 4 weeks after the transplantation, half of them were treated with seven injections of poly(IC). For Ly5.1/5.2 competitive transplantation, equal numbers of unfractionated BM cells from Ly5.1 and Ly5.2 were pooled and co-transplanted into the recipients intravenously through the tail vein. For irradiated setting, lethally irradiated control (*Mx1*-Cre^{-} *Gata2*$^{fl/fl}$) mice were used as recipients. For pre-induction setting, *Mx1*-Cre^{+} *Gata2*$^{fl/fl}$ mice were received four injections of poly(IC) before the transplantation. At 2 days after the last injection, 1×10^6 unfractionated BM cells from each strain were co-transplanted into the recipients along with 2×10^5 unfractionated BM (F1) as support. For post-induction setting, 10×10^6 unfractionated BM cells from each strain were co-transplanted into the recipients without any conditioning and seven injections of poly(IC) were administered at 4 weeks after the transplantation. PB from tail veins was collected and stained with anti-CD45.1 (A20, BioLegend), anti-CD45.2 (104, BioLegend), anti-CD4 (GkK1.5, BioLegend), anti-CD8a (53–6.7, BioLegend), anti-Gr-1 (RB6-8C5, BioLegend), anti-Cd11b (M1/70, BioLegend), and anti-B220 (RA3-6B2, BioLegend) antibodies to monitor the donor contribution. Sixteen to 24 weeks after the transplantation, recipients were sacrificed and donor engraftment in the BM was analyzed.

Gene expression analysis

Total RNA was isolated from FACS sorted LSK cells (RNeasy Micro Kit, no. 74004, QIAGEN) and reverse transcribed (SuperScript III First Strand Synthesis Kit, no. 18080-085, Invitrogen) in the presence of random hexamers. Real-time qPCR were performed using TaqMan Gene Expression Master Mix (Fisher Scientific, no. 4369016) and gene-specific probes (*Cdh19*: Mm01285258_m1, *CD62*l: Mm00441291_m1, *Cxcr4*: Mm01292123_m1, *Hprt*: Mm03024075_m1, *Nav1*: Mm00557902_m1) on a 7900HT Fast Real-Time PCR system (Applied Biosystems). Gene expression levels were normalized to the house keeping gene *Hprt*.

Cell-cycle analysis

Freshly isolated c-KIT-enriched BM cells stained with HSC markers were fixed and permeabilized using a BDCytofix/Cytoperm Fixation /Permeabilization Kit (BD), and stained with anti-Ki67 antibody and DAPI. Cell-cycle status was then determined based on the Ki67 expression and DNA content on FACS LSRII.

Homing assay

Cells were stained with CFSE (C34570; Invitrogen) or SNARF-1 (S-22801; Invitrogen) at a final concentration of 5 μM as described previously (Grassinger and Nilsson, 2011). In brief, samples were stained in phosphate-buffered saline (PBS) supplemented with 0.5% FBS at 37°C. After 20 min, staining was quenched with ice-cold PBS supplemented with 20% FBS, the cells were washed twice with PBS containing 2% FBS. Equal numbers of cells were mixed based on the counting after the staining. Approximately 80,000 of LSK or 1.3×10^6 of Lin^{-} cells from each strain were co-transplanted into the recipients (Ly5.1/5.2 F1) without irradiation. Homing to the BM was measured by the presence of $CFSE^{+}$ and SNARF-1^{+} cells after 12–14 h. DAPI was used to exclude the dead cells. Approximately 10×10^6 of total events were collected by FACS to ensure 50 to 100 positive events. To minimize the size of each sample files, unstained and autofluorescent cell fraction were excluded as an inverted gate.

Statistical analysis

The results are expressed as the mean ± standard deviation for n given samples (representing independent biological repeats). Statistical significance was determined using two-tailed unpaired Student's t test or Mann-Whitney test (*p < 0.05, **p < 0.01, ***p < 0.001, ****p < 0.0001; ns, not significant). For Kaplan-Meier curve, Mantel-Cox test was used. All statistical analyses were performed on Prism 9 (GraphPad).

Data and code availability

All relevant data are available from the corresponding author upon reasonable request.

SUPPLEMENTAL INFORMATION

Supplemental information can be found online at https://doi.org/10.1016/j.stemcr.2021.05.019.

AUTHOR CONTRIBUTIONS

N.M., A.R., J.R. and J.L. planned the study and designed the experiments. N.M. performed the majority of experiments with assistance from A.R. and J.R. N.M. and J.R. analyzed the data. N.M. and J.L. wrote the manuscript with input from the other authors.

ACKNOWLEDGMENTS

We thank Marjo Salminen for providing the *Gata2* conditional knockout mice. This work was funded by grants from the Swedish Research Council, the Swedish Cancer Foundation, the Swedish Pediatric Cancer Foundation, Knut och Alice Wallenbergs Stiftelse, and the European Research Council (ERC) under the European Union's Horizon 2020 research and innovation program (grant agreement no. 648894) to J.L. The work was further supported by the HematoLinné and StemTherapy programs at Lund University.

Received: December 1, 2020
Revised: May 23, 2021
Accepted: May 25, 2021
Published: June 24, 2021

REFERENCES

Akinduro, O., Weber, T.S., Ang, H., Haltalli, M.L.R., Ruivo, N., Duarte, D., Rashidi, N.M., Hawkins, E.D., Duffy, K.R., and Lo Celso, C. (2018). Proliferation dynamics of acute myeloid leukaemia and haematopoietic progenitors competing for bone marrow space. Nat. Commun. *9*, 519.

Bhattacharya, D., Czechowicz, A., Ooi, A.G.L., Rossi, D.J., Bryder, D., and Weissman, I.L. (2009). Niche recycling through division-independent egress of hematopoietic stem cells. J. Exp. Med. *206*, 2837–2850.

Bhattacharya, D., Rossi, D.J., Bryder, D., and Weissman, I.L. (2006). Purified hematopoietic stem cell engraftment of rare niches corrects severe lymphoid deficiencies without host conditioning. J. Exp. Med. *203*, 73–85.

Broxmeyer, H.E., Orschell, C.M., Clapp, D.W., Hangoc, G., Cooper, S., Plett, P.A., Liles, W.C., Li, X., Graham-Evans, B., Campbell, T.B., et al. (2005). Rapid mobilization of murine and human hematopoietic stem and progenitor cells with AMD3100, a CXCR4 antagonist. J. Exp. Med. *201*, 1307–1318.

Busch, K., Klapproth, K., Barile, M., Flossdorf, M., Holland-Letz, T., Schlenner, S.M., Reth, M., Höfer, T., and Rodewald, H.-R. (2015). Fundamental properties of unperturbed haematopoiesis from stem cells in vivo. Nature *518*, 542–546.

Busch, K., and Rodewald, H.-R. (2016). Unperturbed vs. post-transplantation hematopoiesis. Curr. Opin. Hematol. *23*, 295–303.

Cao, X., Wu, X., Frassica, D., Yu, B., Pang, L., Xian, L., Wan, M., Lei, W., Armour, M., Tryggestad, E., et al. (2011). Irradiation induces bone injury by damaging bone marrow microenvironment for stem cells. Proc. Natl. Acad. Sci. U S A *108*, 1609–1614.

Capitano, M.L., Hangoc, G., Cooper, S., and Broxmeyer, H.E. (2015). Mild heat treatment primes human CD34(+) cord blood cells for migration toward SDF-1alpha and enhances engraftment in an NSG mouse model. Stem Cells *33*, 1975–1984.

Chisolm, D.A., Cheng, W., Colburn, S.A., Silva-Sanchez, A., Meza-Perez, S., Randall, T.D., and Weinmann, A.S. (2019). Defining genetic variation in widely used congenic and backcrossed mouse models reveals varied regulation of genes important for immune responses. Immunity *51*, 155–168 e155.

Colvin, G.A., Lambert, J.F., Abedi, M., Hsieh, C.C., Carlson, J.E., Stewart, F.M., and Quesenberry, P.J. (2004). Murine marrow cellularity and the concept of stem cell competition: geographic and quantitative determinants in stem cell biology. Leukemia *18*, 575–583.

Czechowicz, A., Kraft, D., Weissman, I.L., and Bhattacharya, D. (2007). Efficient transplantation via antibody-based clearance of hematopoietic stem cell niches. Science *318*, 1296–1299.

de Pater, E., Kaimakis, P., Vink, C.S., Yokomizo, T., Yamada-Inagawa, T., van der Linden, R., Kartalaei, P.S., Camper, S.A., Speck, N., and Dzierzak, E. (2013). Gata2 is required for HSC generation and survival. J. Exp. Med. *210*, 2843–2850.

Domen, J., Gandy, K.L., and Weissman, I.L. (1998). Systemic overexpression of BCL-2 in the hematopoietic system protects transgenic mice from the consequences of lethal irradiation. Blood *91*, 2272–2282.

Dominici, M., Rasini, V., Bussolari, R., Chen, X., Hofmann, T.J., Spano, C., Bernabei, D., Veronesi, E., Bertoni, F., Paolucci, P., et al. (2009). Restoration and reversible expansion of the osteoblastic hematopoietic stem cell niche after marrow radioablation. Blood *114*, 2333–2343.

Essers, M.A.G., Offner, S., Blanco-Bose, W.E., Waibler, Z., Kalinke, U., Duchosal, M.A., and Trumpp, A. (2009). IFNα activates dormant haematopoietic stem cells in vivo. Nature *458*, 904–908.

Foudi, A., Jarrier, P., Zhang, Y., Wittner, M., Geay, J.-F., Lecluse, Y., Nagasawa, T., Vainchenker, W., and Louache, F. (2006). Reduced retention of radioprotective hematopoietic cells within the bone marrow microenvironment in $CXCR4^{-/-}$ chimeric mice. Blood *107*, 2243–2251.

Grassinger, J., and Nilsson, S.K. (2011). Methods to analyze the homing efficiency and spatial distribution of hematopoietic stem and progenitor cells and their relationship to the bone marrow endosteum and vascular endothelium. Methods Mol. Biol. (Clifton, NJ) *750*, 197–214.

Guo, B., Huang, X., Cooper, S., and Broxmeyer, H.E. (2017). Glucocorticoid hormone-induced chromatin remodeling enhances human hematopoietic stem cell homing and engraftment. Nat. Med. *23*, 424–428.

Haugas, M., Lilleväli, K., Hakanen, J., and Salminen, M. (2010). Gata2 is required for the development of inner ear semicircular ducts and the surrounding perilymphatic space. Dev. Dyn. *239*, 2452–2469.

Heazlewood, S.Y., Oteiza, A., Cao, H., and Nilsson, S.K. (2014). Analyzing hematopoietic stem cell homing, lodgment, and engraftment to better understand the bone marrow niche. Ann. New York Acad. Sci. *1310*, 119–128.

Huang, X., Guo, B., Liu, S., Wan, J., and Broxmeyer, H.E. (2018). Neutralizing negative epigenetic regulation by HDAC5 enhances human haematopoietic stem cell homing and engraftment. Nat. Commun. *9*, 2741.

Kiel, M.J., Yilmaz, O.H., Iwashita, T., Yilmaz, O.H., Terhorst, C., and Morrison, S.J. (2005). SLAM family receptors distinguish hematopoietic stem and progenitor cells and reveal endothelial niches for stem cells. Cell *121*, 1109–1121.

Kühn, R., Schwenk, F., Aguet, M., and Rajewsky, K. (1995). Inducible gene targeting in mice. Science *269*, 1427–1429.

Li, H.S., Jin, J., Liang, X., Matatall, K.A., Ma, Y., Zhang, H., Ullrich, S.E., King, K.Y., Sun, S.-C., and Watowich, S.S. (2016). Loss of c-Kit and bone marrow failure upon conditional removal of the GATA-2 C-terminal zinc finger domain in adult mice. Eur. J. Haematol. *97*, 261–270.

Lim, K.-C., Hosoya, T., Brandt, W., Ku, C.-J., Hosoya-Ohmura, S., Camper, S.A., Yamamoto, M., and Engel, J.D. (2012). Conditional Gata2 inactivation results in HSC loss and lymphatic mispatterning. J. Clin. Invest. *122*, 3705–3717.

Ling, K.-W., Ottersbach, K., van Hamburg, J.P., Oziemlak, A., Tsai, F.-Y., Orkin, S.H., Ploemacher, R., Hendriks, R.W., and Dzierzak, E. (2004). GATA-2 plays two functionally distinct roles during the ontogeny of hematopoietic stem cells. J. Exp. Med. *200*, 871–882.

Lu, R., Czechowicz, A., Seita, J., Jiang, D., and Weissman, I.L. (2019). Clonal-level lineage commitment pathways of hematopoietic stem cells in vivo. Proc. Natl. Acad. Sci. *116*, 1447–1456.

Menendez-Gonzalez, J.B., Vukovic, M., Abdelfattah, A., Saleh, L., Almotiri, A., Thomas, L.-a., Agirre-Lizaso, A., Azevedo, A., Menezes, A.C., Tornillo, G., et al. (2019). Gata2 as a crucial regulator of stem cells in adult hematopoiesis and acute myeloid leukemia. Stem Cell Reports *13*, 291–306.

Mercier, F.E., Sykes, D.B., and Scadden, D.T. (2016). Single targeted exon mutation creates a true congenic mouse for competitive hematopoietic stem cell transplantation: the C57BL/6-CD45.1 STEM mouse. Stem Cell Reports *6*, 985–992.

Migliaccio, A.R., Carta, C., and Migliaccio, G. (1999). In vivo expansion of purified hematopoietic stem cells transplanted in nonablated W/Wv mice. Exp. Hematol. *27*, 1655–1666.

Nie, Y., Han, Y.-C., and Zou, Y.-R. (2008). CXCR4 is required for the quiescence of primitive hematopoietic cells. J. Exp. Med. *205*, 777–783.

Nilsson, S.K., Dooner, M.S., Tiarks, C.Y., Weier, H.U., and Quesenberry, P.J. (1997). Potential and distribution of transplanted hematopoietic stem cells in a nonablated mouse model. Blood *89*, 4013–4020.

Nilsson, S.K., Dooner, M.S., Weier, H.U., Frenkel, B., Lian, J.B., Stein, G.S., and Quesenberry, P.J. (1999). Cells capable of bone production engraft from whole bone marrow transplants in nonablated mice. J. Exp. Med. *189*, 729–734.

Osawa, M., Hanada, K., Hamada, H., and Nakauchi, H. (1996). Long-term lymphohematopoietic reconstitution by a single CD34-low/negative hematopoietic stem cell. Science (New York, NY) *273*, 242–245.

Rodrigues, N.P. (2005). Haploinsufficiency of GATA-2 perturbs adult hematopoietic stem-cell homeostasis. Blood *106*, 477–484.

Schofield, R. (1978). The relationship between the spleen colony-forming cell and the haemopoietic stem cell. Blood cells *4*, 7–25.

Shimoto, M., Sugiyama, T., and Nagasawa, T. (2017). Numerous niches for hematopoietic stem cells remain empty during homeostasis. Blood *129*, 2124–2131.

Smith, L.G., Weissman, I.L., and Heimfeld, S. (1991). Clonal analysis of hematopoietic stem-cell differentiation in vivo. Proc. Natl. Acad. Sci. U S A *88*, 2788–2792.

Sugiyama, T., Kohara, H., Noda, M., and Nagasawa, T. (2006). Maintenance of the hematopoietic stem cell pool by CXCL12-CXCR4 chemokine signaling in bone marrow stromal cell niches. Immunity *25*, 977–988.

Sun, J., Ramos, A., Chapman, B., Johnnidis, J.B., Le, L., Ho, Y.-J., Klein, A., Hofmann, O., and Camargo, F.D. (2014). Clonal dynamics of native haematopoiesis. Nature *514*, 322–327.

Till, J.E., and Mc, C.E. (1961). A direct measurement of the radiation sensitivity of normal mouse bone marrow cells. Radiat. Res. *14*, 213–222.

Tomita, Y., Sachs, D.H., and Sykes, M. (1994). Myelosuppressive conditioning is required to achieve engraftment of pluripotent stem cells contained in moderate doses of syngeneic bone marrow. Blood *83*, 939–948.

Tsai, F.Y., Keller, G., Kuo, F.C., Weiss, M., Chen, J., Rosenblatt, M., Alt, F.W., and Orkin, S.H. (1994). An early haematopoietic defect in mice lacking the transcription factor GATA-2. Nature *371*, 221–226.

Velasco-Hernandez, T., Säwén, P., Bryder, D., and Cammenga, J. (2016). Potential pitfalls of the *Mx1-Cre* system: implications for experimental modeling of normal and malignant hematopoiesis. Stem Cell Reports *7*, 11–18.

Ventura, A., Kirsch, D.G., McLaughlin, M.E., Tuveson, D.A., Grimm, J., Lintault, L., Newman, J., Reczek, E.E., Weissleder, R., and Jacks, T. (2007). Restoration of p53 function leads to tumour regression in vivo. Nature *445*, 661–665.

Wang, Z., Li, G., Tse, W., and Bunting, K.D. (2009). Conditional deletion of STAT5 in adult mouse hematopoietic stem cells causes loss of quiescence and permits efficient nonablative stem cell replacement. Blood *113*, 4856–4865.

Waskow, C., Madan, V., Bartels, S., Costa, C., Blasig, R., and Rodewald, H.-R. (2009). Hematopoietic stem cell transplantation without irradiation. Nat. Methods *6*, 267–269.

Waterstrat, A., Liang, Y., Swiderski, C.F., Shelton, B.J., and Van Zant, G. (2010). Congenic interval of CD45/Ly-5 congenic mice contains multiple genes that may influence hematopoietic stem cell engraftment. Blood *115*, 408–417.

Westerhuis, G., van Pel, M., Toes, R.E., Staal, F.J., and Fibbe, W.E. (2011). Chimerism levels after stem cell transplantation are primarily determined by the ratio of donor to host stem cells. Blood *117*, 4400–4401.

Zhang, M., Yin, L., Zhang, K., Sun, W., Yang, S., Zhang, B., Salzman, P., Wang, W., Liu, C., Vidyasagar, S., et al. (2012). Response patterns of cytokines/chemokines in two murine strains after irradiation. Cytokine *58*, 169–177.

Stem Cell Reports, Volume *16*

Supplemental Information

Uncoupling key determinants of hematopoietic stem cell engraftment through cell-specific and temporally controlled recipient conditioning

Natsumi Miharada, Anna Rydström, Justyna Rak, and Jonas Larsson

Supplemental Information

Uncoupling key determinants of hematopoietic stem cell engraftment through cell specific and temporally controlled recipient conditioning

Natsumi Miharada *et al.*

Figure S1, related to Figure 1. Conditional *Gata2* deletion depletes phenotypic and functional HSCs

(A) Genomic PCR results with unfractionated BM from ER-Cre^{+} *Gata2*$^{fl/fl}$ and control (ER-Cre^{-} *Gata2*$^{fl/fl}$) mice administrated with tamoxifen and harvested at 1 week after the last injection. (B) Representative FACS plots showing LSK compartment at 1 day after the last tamoxifen injection. (C) Frequency (left) and cell number (right) of LSK from ER-Cre^{+} *Gata2*$^{fl/fl}$ and control mice (ER-Cre^{-} *Gata2*$^{fl/fl}$) at 1-7 day after the last tamoxifen injection. Data are pooled from three independent experiments (n = 10). (D and E) BM (Ly5.2) from ER-Cre^{+} *Gata2*$^{fl/fl}$ mice that received 5 injections of tamoxifen or vehicle were transplanted together with WT BM (Ly5.1) cells into irradiated recipients in a competitive manner (n = 6-7). Frequency of Ly5.2 cells in PB (D) and BM (E) after the transplantation. Representative FACS plots showing the contribution from each donor at 20 weeks after transplantation (n = 6-7). n represents independent biological replicates, except in D and E where n represents technical replicates (recipient mice) from 3 independent biological repeats (donor mice).

Figure S2, related to Figure 2. *Gata2* deletion enables efficient engraftment of transplanted HSCs

(A and B) Frequency of donor derived cells within each lineages in PB after the transplantation, corresponding to Figure 2B and 2E respectively. (C) 70,000 LSK cells were transplanted into ER-Cre^{+} *Gata2*$^{fl/fl}$ or control (ER-Cre^{-} *Gata2*$^{fl/fl}$) mice. 4 weeks after the transplantation, recipients were treated with tamoxifen for 5 injections. (D) Donor contribution in PB was monitored for up to 22 weeks after the last tamoxifen injection (n = 3-4). (E) Frequency of donor in BM LSK at 22 weeks after the last tamoxifen injection (n = 3-4). n represents independent biological replicates.

Figure S3, related to Figure 2. Detection of donor engraftment in non-conditioned recipients

(A) 320,000 LSK cells from WT (Ly5.2) were transplanted into WT recipients (Ly5.1/5.2 F1) without irradiation. After 4 weeks, total BM cells of primary recipients were harvested, and 5 million cells were further transplanted into lethally irradiated secondary WT recipients (Ly5.1/5.2 F1). (B) Frequency of donor derived cells in the BM HSC at 4 weeks after the primary transplantation (n = 3). FACS plots showing the gating strategy for the non-transplanted control mouse (above) and primary recipient (below). (C) Frequency of initial donor derived cells in the peripheral blood of secondary recipients (BM from 3 primary recipients was transplanted to 2 secondary recipients each). n represents independent biological replicates.

Figure S4, related to Figure 4. Advantage in homing is associated with higher engraftment potential in non-irradiated recipients

(A) Relative mRNA expression of indicated genes normalized to *Hprt* (LSK n = 9, HSC n = 5). (B) As a reference for the initial ratio of Ly5.1 (labelled with CFSE) and Ly5.2 (labelled with SNARF-1) cells before the transplantation, some residues of the transplanted cells were kept on ice and analyzed at the same time with the homed cells to the BM in the recipients. (C) Gating strategy to record statistically relevant numbers of CFSE and SNARF-1 positive cells homed to the bone marrow. After doublets and dead cell exclusion, unstained and autofluorescent cells were excluded as an inverted gate. n represents independent biological replicates.

Figure S1.

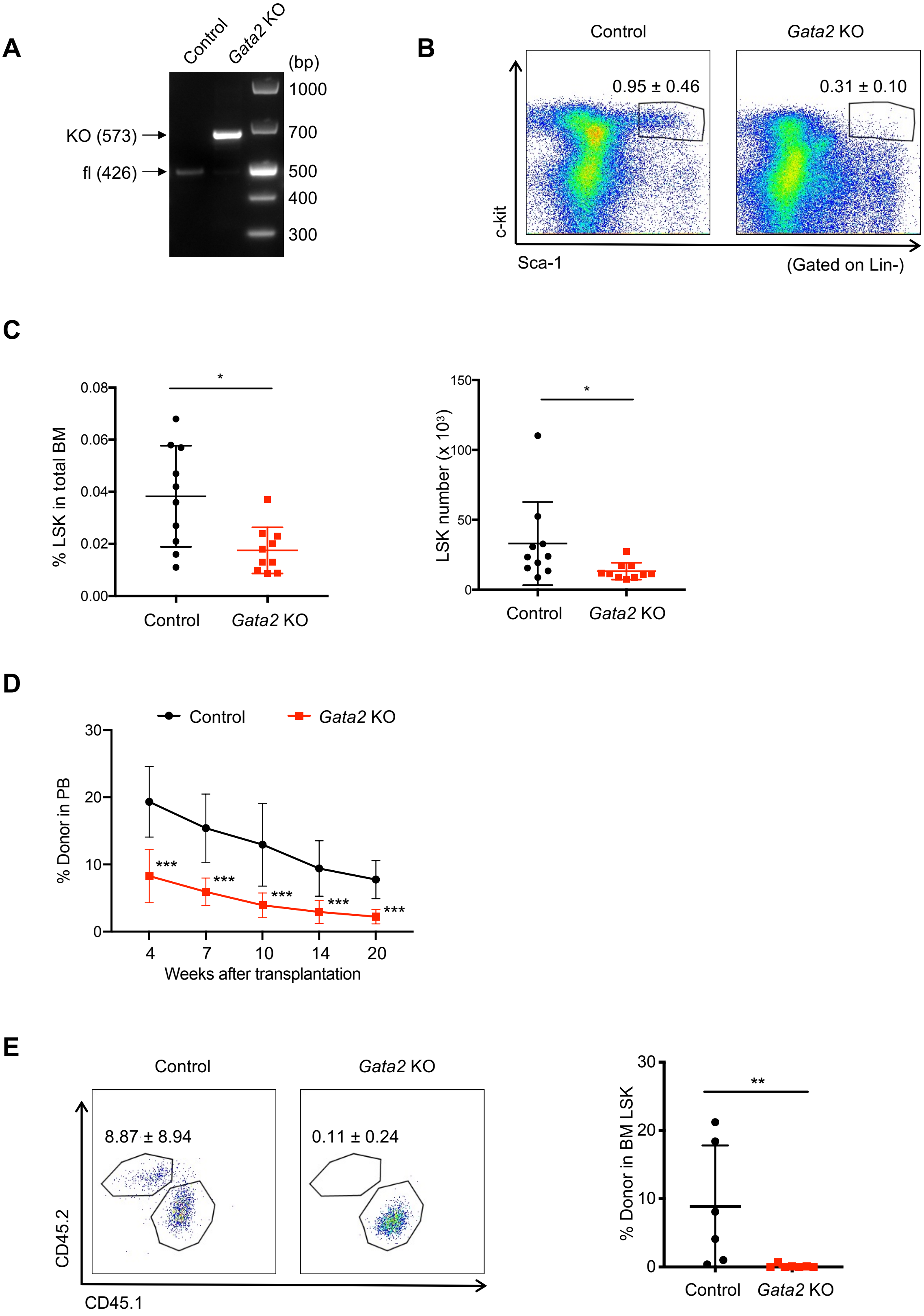

A
Control
Gata2 KO
(bp)
1000
700
500
400
300
KO (573)
fl (426)
B
Control
Gata2 KO
0.95 ± 0.46
0.31 ± 0.10
c-kit
Sca-1
(Gated on Lin-)
C
% LSK in total BM
0.08
0.06
0.04
0.02
0.00
*
Control
Gata2 KO
LSK number (x 10³)
150
100
50
0
*
Control
Gata2 KO
D
Control
Gata2 KO
% Donor in PB
30
20
10
0
***
***
***
***
***
4
7
10
14
20
Weeks after transplantation
E
Control
Gata2 KO
8.87 ± 8.94
0.11 ± 0.24
CD45.2
CD45.1
% Donor in BM LSK
30
20
10
0
**
Control
Gata2 KO

Figure S2.

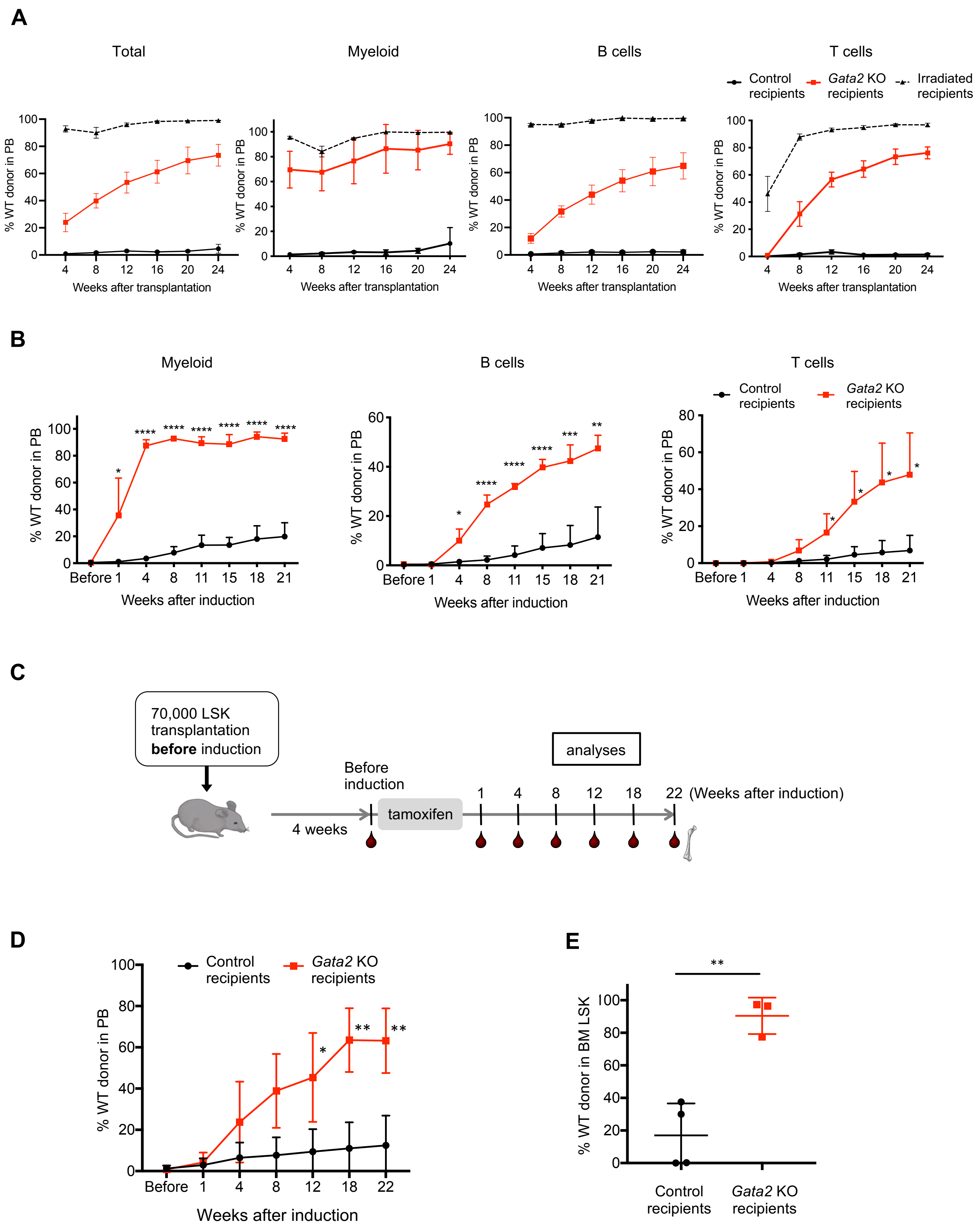

A
Total
Myeloid
B cells
T cells
Control recipients
Gata2 KO recipients
Irradiated recipients
% WT donor in PB
Weeks after transplantation
B
Myeloid
B cells
T cells
Control recipients
Gata2 KO recipients
% WT donor in PB
Weeks after induction
C
70,000 LSK transplantation **before** induction
4 weeks
Before induction
tamoxifen
analyses
1 4 8 12 18 22 (Weeks after induction)
D
Control recipients
Gata2 KO recipients
% WT donor in PB
Weeks after induction
E
% WT donor in BM LSK
Control recipients
Gata2 KO recipients

Figure S3.

A

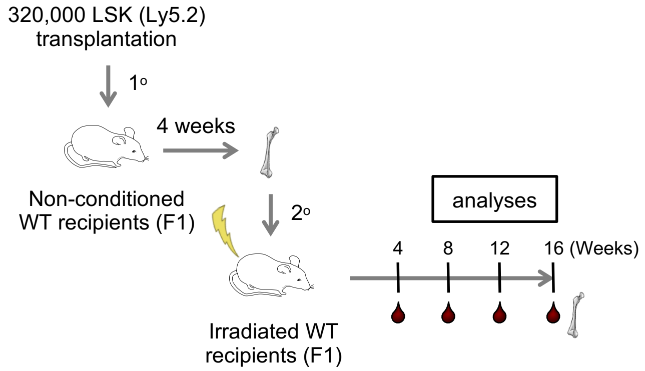

B

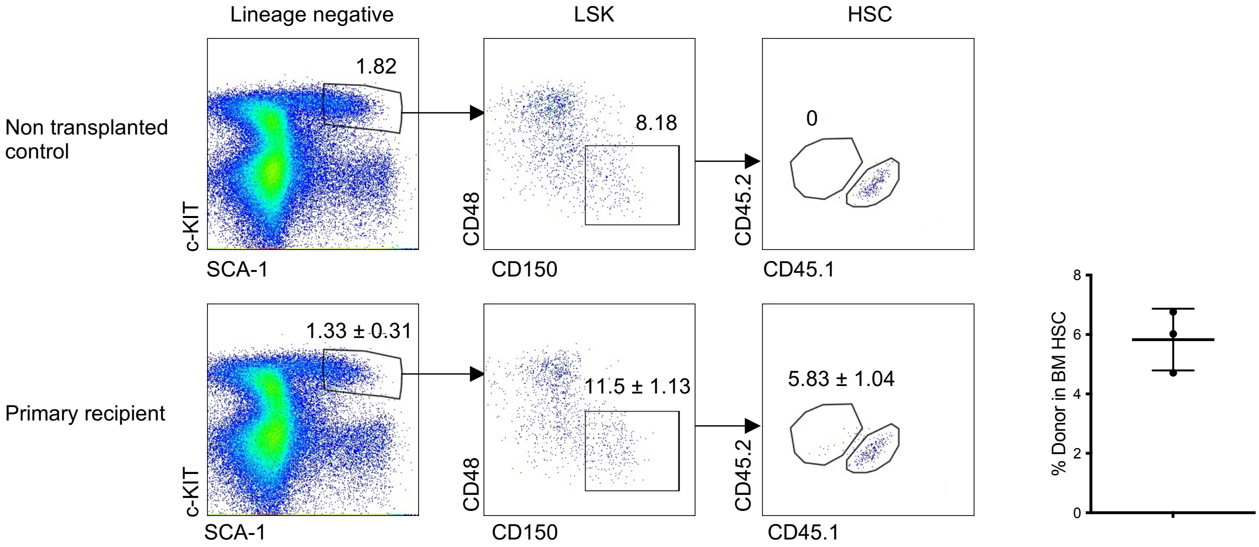

C

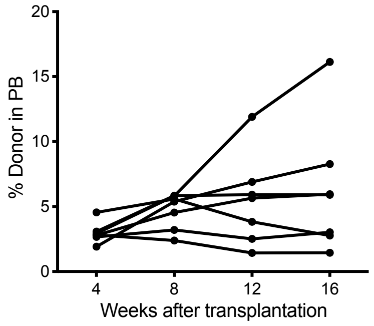

Figure S4.

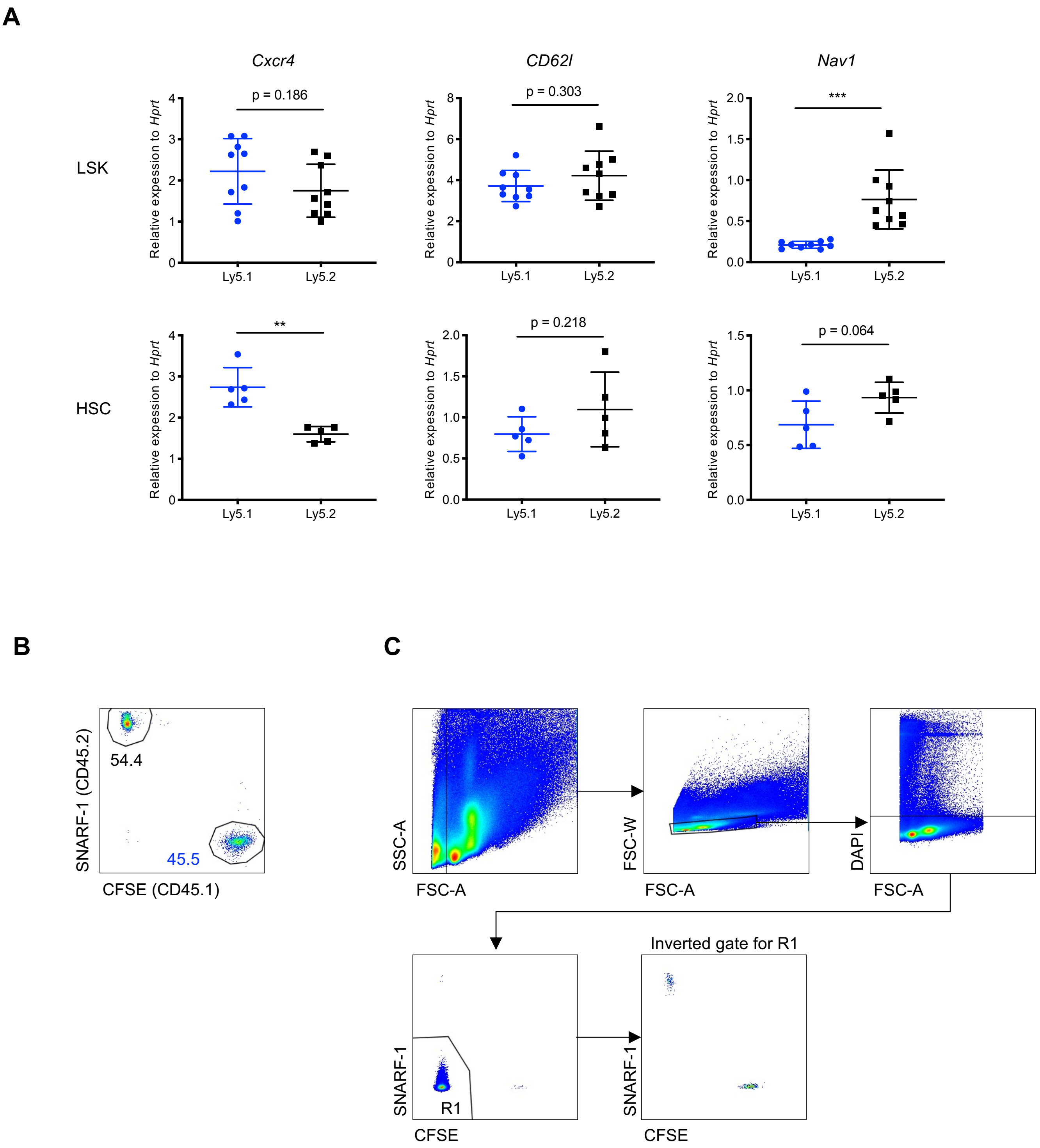
A
Cxcr4
CD62l
Nav1
LSK
HSC
Relative expession to Hprt
p = 0.186
p = 0.303
***
**
p = 0.218
p = 0.064
Ly5.1
Ly5.2
B
C
54.4
45.5
SNARF-1 (CD45.2)
CFSE (CD45.1)
SSC-A
FSC-A
FSC-W
DAPI
SNARF-1
CFSE
R1
Inverted gate for R1
